# Differences in food consumption of the Brazilian population by race/skin color in 2017–2018

**DOI:** 10.11606/s1518-8787.2023057004000

**Published:** 2023-02-08

**Authors:** Janaína Calu Costa, Amanda Cristina da Silva de Jesus, Juliana Giaj Levra de Jesus, Mariana Ferreira Madruga, Thays Nascimento Souza, Maria Laura da Costa Louzada

**Affiliations:** I Universidade Federal de Pelotas Centro Internacional para Equidade em Saúde Programa de Pós-Graduação em Epidemiologia Pelotas RS Brasil Universidade Federal de Pelotas. Centro Internacional para Equidade em Saúde. Programa de Pós-Graduação em Epidemiologia. Pelotas, RS, Brasil; II Universidade de São Paulo Faculdade de Saúde Pública Programa de Pós-Graduação em Nutrição em Saúde Pública São Paulo SP Brasil Universidade de São Paulo. Faculdade de Saúde Pública. Programa de Pós-Graduação em Nutrição em Saúde Pública. São Paulo, SP, Brasil; III Universidade de São Paulo Faculdade de Medicina Programa de Pós-Graduação em Saúde Coletiva São Paulo SP Brasil Universidade de São Paulo. Faculdade de Medicina. Programa de Pós-Graduação em Saúde Coletiva. São Paulo, SP, Brasil.; IV Universidade de São Paulo Núcleo de Pesquisas Epidemiológicas em Nutrição e Saúde São Paulo SP Brasil Universidade de São Paulo. Núcleo de Pesquisas Epidemiológicas em Nutrição e Saúde. São Paulo, SP, Brasil

**Keywords:** Diet, Food, and Nutrition, Race Factors, Socioeconomic Factors, Nutrition Surveys

## Abstract

**OBJECTIVE:**

To evaluate food consumption in Brazil by race/skin color of the population.

**METHODS:**

Food consumption data from the *Pesquisa de Orçamentos Familiares* (POF – Household Budget Survey) 2017–2018 were analyzed. Food and culinary preparations were grouped into 31 items, composing three main groups, defined by industrial processing characteristics: 1 – *in natura*/minimally processed, 2 – processed, and 3 – ultra-processed. The percentage of calories from each group was estimated by categories of race/skin color – White, Black, Mixed-race, Indigenous, and Yellow– using crude and adjusted linear regression for gender, age, schooling, income, macro-region, and area.

**RESULTS:**

In the crude analyses, the consumption of *in natura*/minimally processed foods was lower for Yellow [66.0% (95% Confidence Interval 62.4–69.6)] and White [66.6% (95%CI 66.1–67.1)] groups than for Blacks [69.8% (95%CI 68.9–70.8)] and Mixed-race people [70.2% (95%CI 69.7–70.7)]. Yellow individuals consumed fewer processed foods, with 9.2% of energy (95%CI 7.2–11.1) whereas the other groups consumed approximately 13%. Ultra-processed foods were less consumed by Blacks [16.6% (95%CI 15.6–17.6)] and Mixed-race [16.6% (95%CI 16.2–17.1)], with the highest consumption among White [20.1% (95%CI 19.6–20.6)] and Yellow [24.5% (95%CI 20.0–29.1)] groups. The adjustment of the models reduced the magnitude of the differences between the categories of race/skin color. The difference between Black and Mixed-race individuals from the White ones decreased from 3 percentage points (pp) to 1.2 pp in the consumption of *in natura*/minimally processed foods and the largest differences remained in the consumption of rice and beans, with a higher percentage in the diet of Black and Mixed-race people. The contribution of processed foods remained approximately 4 pp lower for Yellow individuals. The consumption of ultra-processed products decreased by approximately 2 pp for White and Yellow groups; on the other hand, it increased by 1 pp in the consumption of Black, Mixed-race, and Indigenous peoples.

**CONCLUSION:**

Differences in food consumption according to race/skin color were found and are influenced by socioeconomic and demographic conditions.

## INTRODUCTION

The Brazilian population is characterized by a great ethnic-racial diversity, which is reflected in the culture and identity of the country^1^. This diversity, however, is associated with important inequities in the living and health conditions of the population, since some groups are in a situation of greater vulnerability, due to the socio-historical processes that contributed to their segregation and marginalization, especially those identified racially, such as the Black, Mixed-race, and Indigenous population^2,3^.

The economic and social disadvantages faced by these groups reflect the mechanisms by which racism contributes to racial inequalities in health, establishing it as an important social determinant of health^4,5^. Thus, racism significantly influences survival conditions, access to services, and behaviors, including those related to food.

Similar to national estimates that mask subnational inequalities, analyzing the health of the population by other social dimensions is insufﬁcient to identify racial inequalities. The use of information about race/skin color is a way to give statistical visibility to the groups while enable measures that meet their different demands to be taken. Despite the usual collection of skin color or race information in official population surveys and the mandatory field in health forms and information systems in Brazil, few studies on food consumption include analyses of inequalities by categories of race/skin color^6^.

Recommendations of the Dietary Guidelines for the Brazilian Population and its shaping principles include the understanding that food is more than nutrient intake, reflecting social contexts, patterns and dietary traditions, and the impact of production and consumption^14^. The recommendations are based on the NOVA classification, which groups food into categories according to the extent and purpose of industrial processing and which has proven useful for understanding the epidemiology of diseases and the impacts of consumption on food systems worldwide^14^.

Trends in Brazil show an increase in the consumption of ultra-processed foods and concomitant decline in the consumption of *in natura* and minimally processed foods^17,18^. Estimates, however, are usually presented stratified by gender, age, region, area, and income ranges, but not by categories of race or sink color of the population, hiding possible inequalities between these groups^17,18^.

To contribute to fill this gap, this study aimed to evaluate food consumption in Brazil in 2017–2018, according to characteristics of industrial processing and by the population race/skin color.

## METHODS

### Data Source and Sampling

The data analyzed are the personal food consumption module of the *Pesquisa de Orçamentos Familiares* (POF – Household Budget Survey), conducted by the Brazilian Institute of Geography and Statistics (IBGE) between July 2017 and July 2018^17^. A complex two-stage sampling process was carried out, by grouping of census tracts with geographic and socioeconomic stratification and subsequent drawn in the first stage, followed by drawing the households belonging to the sectors selected in the second stage^17^.

### Food Consumption

Information regarding individual food consumption was collected in a subsample of 20,112 households and reported by residents aged 10 years or older^17^. To the 46,164 individuals selected for the consumption module, 24-hour dietary recalls were applied on two non-consecutive days^17^.

The amount of each food or drink recorded in the recalls was transformed into grams or milliliters and converted into energy (kilocalories, kcal) based on the Brazilian Food Composition Table^19^. Food and culinary preparations were grouped based on the NOVA classification, which classifies food items according to characteristics of industrial processing^20^. The categorization was adapted according to the methodology used by Louzada et al. (2015), in which the preparations are not decomposed into ingredients, prevailing the characteristics of the main items reported^21^. The three main groups have 31 subgroups created from foods consumed in isolation or in culinary preparations with multiple ingredients, as described in the Chart.

**Chart:** Classification of foods reported in the *Pesquisa de Orçamentos Familiares* (POF – Household Budget Survey) 2017–2018 according to groups and subgroups of the NOVA food classification.

NOVA Groups		Foods
In natura or minimally-processed food items and culinary ingredients		
1	Rice	White rice, parboiled, needle, whole grain, 3 grains, and 7 grains Rice-based preparations (rice dumpling, coconut rice, milk rice, risotto)
2	Beef and pork	Beef or pork (steak, filet mignon, muscle, beef rib, ground beef, pork rib, ham, loin)
		Red meat-based preparations (kafta, shredded beef, roast beef, roasted ham, grilled steak, roast pig)
3	Beans	Black beans, pinto, green, purple mulatto, black-eyed pea, pigeon pea
		Bean-based preparations (bean soup and bean broth)
4	Poultry meat	Chicken, turkey, or duck meat
		Poultry-based preparations (chicken, turkey or roast duck, country chicken, grilled chicken fillet)
5	Fruits and 100% fruit juice	Raw or cooked fruits, fruit salad
		100% fruit juice
6	Pasta	Variety of types of pasta, gnocchi, pancake
		Preparations with pasta (pasta with cheese, pasta with garlic and oil, pasta with white sauce, gratin pasta)
7	Vegetables	Raw, sautéed, fried, roasted or cooked vegetables, and greens
		Vegetable-based preparations (vinaigrette, salads and assorted soups, quibebe, caponata, mixed cooked vegetables, spinach cream, vegetarian kibbeh, ratatouille)
8	Tubers and roots	English or sweet potatoes, yams, cassava or manioc or yuca, arracacha
		Puree-like preparations
9	Eggs	Chicken and quail eggs
		Egg-based preparations (scrambled eggs, soft eggs, omelette, soufflé, eggnog, pasta with eggs)
10	Cassava flour	Cassava flour, fermented cassava flour, tapioca
		Cassava flour-based preparations (plain fried flour, tapioca flour, sweet tapioca pearls)
11	Corn, oats, and wheat (excluding flour)	Quinoa, wheat, corn
		Preparations such as couscous, corn cream, polenta, cornmeal soup
12	Fish	Saltwater or freshwater fish
		Fish-based preparations (sashimi, fish with shrimp, fish gratin, fish stew, fish tartare)
13	Coffees and Teas	Teas made from various herbs (chamomile, lemon grass, black, etc.), mate, chimarrão, tereré
		Coffees (espresso, carioca, with milk, decaffeinated)
14	Mixed preparations of rice/pasta/flours or other cereals + beef/pork/poultry or fish/seafood + tubers/beans/vegetables/greens or preparations with *in natura* or minimally processed foods^a^	Steak rolls, pot meat, feijoada, rib with vegetables, sun meat with manioc, pork with vegetables
		Assorted fish stew, shrimp bobó, acarajé, roasted fish with vegetables, temaki
		Milanesa steak, hamburger, pasta with meat, grinded cassava flour with sun dried meat
		Rice and beans, milanesa vegetables, Greek rice, São Paulo couscous, tabbouleh, eggplant lasagna
		Rice with chicken, brown sauce rice, chicken parmesan
		Tuna couscous, fish creamy manioc, shrimp risotto, rice with seafood
		Cuxá rice, seafood lasagna
		Baião-de-dois, arrumadinho, steak rolls, angu with ground beef and tomato
		Chicken salad with mayonnaise, rice with chicken and pequi, cabbage cigar with chicken
		Strogonoff, mayonnaise salad, acai with granola, rice or pasta with sausage or cured pork sausage, legumes with cured pork sausage, shrimp with creamy curd
15	Homemade desserts	Cakes, breads, pies, and other sweet desserts (simple cakes, ambrosia, coconut candy, Brazilian corn pudding, Brazilian rice pudding, caramelized banana, condensed milk dessert with cashews, Brazilian coconut egg custard)
		Sweet cakes and pies with fresh or minimally processed and ultra-processed foods (milk pudding, Brazilian prune coconut candy, pineapple delight, carrot cake with chocolate filling)
16	Other	Seafood (shrimp, octopus, squid, shellfish, roe)
		Other meats (goat, goatling, sheep, paca, alligator, capybara, tortoise, lamb, and other animals)
		Natural yogurt (kefir, curd, natural yogurt and skimmed yogurt)
		Other flours (oatmeal, copioba or corn, porridge, guarana powder, vatapá, Moroccan couscous)
		Offal (bovine, chicken, goat, pig viscera) and preparations like sarapatel, dobradinha stew, sarrabulho
		Milk (cow or goat, whole, semi-skimmed or skimmed, powdered or liquid, with or without lactose), cream
		Breads and salty pies (homemade bread, quiche, assorted pies)
		Other legumes (peas, broad bean, chickpeas, lentils, soybeans, soy meat and protein)
		Nuts and seeds (includes peanuts): Chestnuts, almond, hazelnut, peanuts, pupunha, buriti, sesame, flax
		Fungi (mushrooms *in natura*)
		Sugars, oils (olive oil, soybean oil, corn, coconut), butter, lard, coconut milk, vinegar and salt
		Starch (tapioca with fillings, tapioca couscous, tapioca pearls in red wine)
		Water
**Processed food items**		
17	French bread and sandwiches	French bread or fermented cassava flour bread or wheat bread (single or whole), bruschetta
		Bread with butter or sandwich with processed breads and fillings of *in natura* or minimally processed foods or processed foods (bread with roast beef, bread with sardines, bread with cheese, bread with egg, bread with chicken or meat)
18	Cheeses	Sandwich cheese, mozzarella cheese, ricotta, gorgonzola, coalho
19	Salted/dried/smoked/cured meats	Jerked meat, sun dried meat, bacon, jabá, parma ham, fried pork skin
20	Beers and wines	Beer, draft beer, wines, sparkling wine, sake, and drinks with these beverages
21	Other	Preserved greens/vegetables (sauerkraut, dried tomatoes, olives, heart of palm, pickles) and mushrooms
		Preserved fruits (guava, banana, pumpkin jam, fruits in sweet syrup, candied fruit candies)
		Preserved legumes (canned corn, canned peas, peanut butter)
		Tomato sauce
		Preserved fish (canned sardines, canned tuna, canned salmon, cod)
**Ultra-processed food items**		
22	Salted crackers and chips	Salted biscuit, salted doughnut, ham flavored snack, packaged snack (potato chips, bacon chips, spicy peanuts, light popcorn)
23	Sweet cookies and baked goods	Doughnut, cookies with filling, sweet manioc flour biscuit, waffle biscuit, and others
		Sweet breads, rolls, yellow custard Berliner, panettone, muffin, croissant with sweet filling
24	Sausages	Beef or fish burger, chicken steak, sausage, cured pork sausage, mortadella, salami, ham, turkey breast, meat pâté, Brazilian calabresa sausage
25	Sweets	Ice cream, popsicle, ice cream in the cone, milk shake, freezie, yogurt-based ice cream
		Chocolate, candy and other sweets (milk or semisweet or white chocolate tablet, chocolate powder, chewing gum, caramel bullet, coconut bullet, gummy candy)
		Other ultra-processed sweets (marshmallow, milk candy, chocolate truffle, Brazilian coconut cheese custard, jam, cereal bar, marron glacé, light and diet sweets, Brazilian coconut marshmallow, had coconut candy, peanut jam)
26	Soft drinks	Soft drinks (including light or diet)
27	Bread, fried and baked snacks, and fast food dishes	Fast food-like snacks (Assorted sandwiches with hamburger, hot dog, wrap, sandwiches with miscellaneous fillings, cheese-egg sandwich, cheese-chicken sandwich)
		Hamburger bread, processed tube bread, corn bread, rye bread, garlic bread, light and diet breads, toasts
		Sandwiches with ultra-processed food filling (cheese and ham, bread with mortadella, salami sandwich)
		Snacks (potato bread, Brazilian chicken croquettes, sfiha, croquette, oven pastry, cheese ball)
		Salty or sweet pizza
28	Yogurts and dairy drinks	Flavored milk, fermented or chocolate milk, chocolate drink, cappuccino coffee, flavored yogurt, concentrated food shake)
29	Artificial juice and other non-alcoholic beverages	Artificial juices and refreshments and non-alcoholic beverages (soy milk, isotonic drink, ready-made teas, non-alcoholic beer and wine, energy drink)
30	Ready-to-eat or semi-ready dishes	Ready-made pasta meals (yakissoba, stuffed cannelloni, stuffed rondele, ready-made lasagna) and instant noodles
31	Other	Cheese cream, processed curd, cream cheese, margarine
		Breakfast cereals (granola, corn flakes with sugar, *farinha láctea*)
		Distilled alcoholic beverages (cachaça, rum, vodka, whisky, cognac, liqueur and drinks with these beverages)
		Industrialized sauces (ketchup, soy sauce, mustard, tartar sauce, salad dressing, light mayonnaise) and heavy cream
		Supplements (protein supplement, vitamins, minerals, dietary supplement, barley powder)

^a^ These preparations may contain ultra-processed ingredients.

The group of *in natura* or minimally processed foods includes items obtained directly from plants or animals and foods that have undergone some process of removal of unwanted parts, drying, pasteurization, freezing, refinement, fermentation, among others, which do not include the addition of substances to the original food^20^. Examples of foods included in this group are: rice and other cereals, beans, meats, fruits and 100% fruit juices, leafy greens, roots and tubers, eggs, pasta, teas and coffee, and flours. Culinary preparations based on one or more *in natura* or minimally processed foods, such as mixed rice, meat and vegetables preparations and homemade desserts, were also included.

Processed foods are products based on *in natura* or minimally processed foods to which one or more ingredients have been added, such as salt or sugar, oil, vinegar, or other culinary substance, such as salted meats, breads made of flour, salt and water and cheeses made of milk and salt^20^. Preparations that combine more than one processed food were also included in this group, such as sandwiches made with freshly made unpackaged bread (“*pão francês*”).

The third group, of ultra-processed foods, includes industrial formulations typically developed from parts of food or from substances synthesized in laboratory, made from numerous ingredients such as sugars and syrups, refined starches, oils and fats, protein isolates, as well as remains from intensively raised animals^20^. *In natura* or minimally processed ingredients represent reduced or null portions in the list of ingredients of ultra-processed foods. For attractiveness, combinations of flavorings, dyes, emulsifiers, thickeners, and other additives that modify sensory characteristics are used. In this group are mass-produced packaged breads, cookies and snacks, sausages, sweets (ice cream, chocolates, candies), soft drinks, ready-to-eat or frozen meals, fast food sandwiches, milk drinks, and artificial juices.

### Data Analysis

Individual food intake was adjusted for intrapersonal variability, using the Multiple Source Method (MSM)^22,23^. From these estimates of adjusted habitual consumption, the average percentage of calories from each of the food groups was calculated for the entire Brazilian population and according to categories of race/skin color, which correspond to the self-declaration of the interviewed population, from choosing one of the five options: White, Black, Mixed-race (*pardo* in Brazilian Portuguese), Indigenous, or Yellow (asked as *amarelo,* meaning yellow and indicates those of some East Asian ancestry). The energy percentage from each of the food groups by race/skin color category was estimated using crude linear regression models as well as adjusted for gender, age group (adolescent, adult, and older adults), quintiles of monthly *per capita* family income in Brazilian reais, quintiles of completed schooling years adjusted for age, geographic macro-region (Midwest, Northeast, North, Southeast, and South) and area of residence (urban or rural).

The estimates considered the complex sample design of the survey and its expansion factors, which allow us to extrapolate the results to the entire Brazilian population. Estimates and respective confidence intervals (95%CI) are presented for the race/skin color categories for all food groups and subgroups. The analyses were performed in *Stata*^®^ software version 14 (College Station, TX: StataCorp LP).

## RESULTS

Among the individuals who responded to the POF food consumption module, 41 did not report race/skin color and, of the others, 44.9% were self-declared Mixed-race, 43.1% White, 10.8% Black, 0.7% Yellow, and 0.4% Indigenous (Table 1).

**Table 1:** Distribution of the Brazilian population and average daily caloric intake, according to race/skin color. Brazil, 2017–2018.

Race/skin color	Frequency in the sample		Average energy intake	
	%	95%CI	kcal/day	95%CI
Mixed-race	44.9	44.0–45.9	1,721.1	1,690.2–1,751.9
White	43.1	42.1–44.2	1,761.5	1,745.6–1,777.4
Black	10.8	10.2–11.5	1,723.9	1,634.3–1,813.5
Yellow	0.7	0.5–0.9	1,756.9	1,742.1–1,771.7
Indigenous	0.4	0.3–0.6	1,713.9	1,618.1–1,809.6
**Total**	**100**	-	**1,754.6**	**1,743.5–1,765.8**

The average energy intake for the Brazilian population was 1,754.6 kcal/day, ranging from 1,713.9 kcal/day among the Indigenous to 1,761.5 kcal/day among the White population (Table 1). Mixed-race, Black, and Yellow groups consumed, on average, 1,721.1 kcal, 1,723.9 kcal, and 1,756.9 kcal/day, respectively. The Figure shows the energy contribution of each of the three major food groups in Brazil and stratified by race/skin color. Nationally, this consumption was characterized by the major participation of *in natura* and minimally processed foods, which corresponded to more than 68% of daily calories. Next, are the ultra-processed (18.2%) and processed foods (13.2%).

**Figure:**
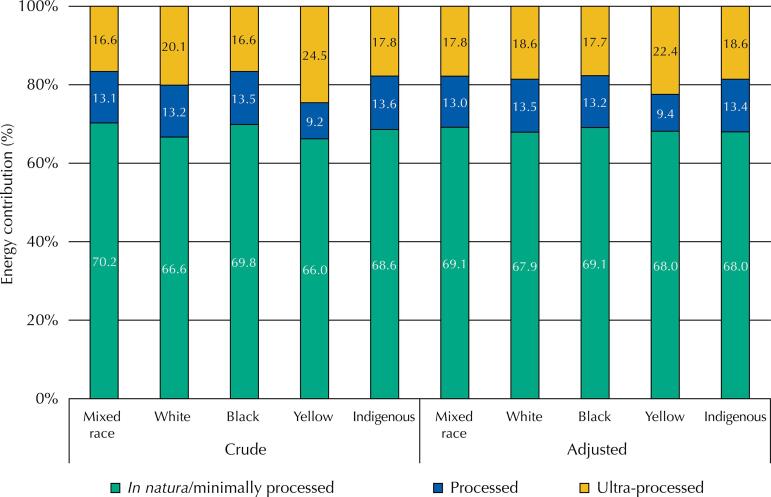
Average daily energy contribution (% kcal/day) of the three food groups, according to characteristics of industrial processing by race/skin color of individuals aged 10 years or older. Crude and adjusted analyses for gender, age, income, schooling, macro-region, and area of residence. Brazil, 2017–2018.

According to the crude analyses, the consumption of *in natura* and minimally processed foods was lower for the Yellow [66.0% (95%CI 62.4–69.6)] and White [66.6% (95%CI 66.1–67.1)] groups, whereas it contributed to approximately 70% of the energy consumed by the Black [69.8% (95%CI 68.9–70.8)] and Mixed-race [70.2% (95%CI 69.7–70.7)] populations. The relative energy contribution of this group for Indigenous was 68.6% (95%CI 64.6–72.6), with no statistical difference from the others.

Regarding processed foods, the Mixed-race [13.1% (95%CI 12.8–13.5)], Black [13.5% (95%CI 12.9–14.2)], White [13.2% (95%CI 12.9–13.5)], and Indigenous [13.6% (95%CI 11.1–16.2)] categories showed no statistical differences. The lowest consumption of foods from this group was observed in the Yellow population, for whom the energy participation was 9.2% (95%CI 7.2%–11.1%), significantly different from the other categories.

Ultra-processed foods had lower participation in the diet of Black [16.6% (95%CI 15.6–17.6)] and Mixed-race people [16.6% (95%CI 16.2–17.1)], slightly lower than in the Indigenous population’s consumption, with 17.8% (95%CI 14.6–21.0). On the other hand, the largest contributions were in the White, with 20.1% (95%CI 19.6–20.6), and Yellow, with 24.5% (95%CI 20.0–29.1) groups.

After adjusting for socioeconomic and demographic variables, the differences observed between the categories of race/skin color decreased in the three food and culinary preparations groups, especially in the ultra-processed food group.

Still, despite the reduction in the magnitude of the differences, the adjusted analyses indicate that White individuals [67.9% (95%CI 67.4–68.3)] were the ones who consumed less *in natura* and minimally processed foods, followed by the Yellow [68.0% (95%CI 65.0–71.1)] and Indigenous people [68.0% (95% CI 64.6–71.4)]. The highest consumption among Black [69.1% (95%CI 68.2–70.0)] and Mixed-race people [69.1% (95%CI 68.6–69.6)] remained after adjustment. The difference between both Black and Mixed-race people to White people decreased from more than 3 percentage points (pp) to 1.2 pp.

The share of processed foods stayed approximately 4 pp lower among the Yellow population [9.4% (95%CI 7.3–11.4)] compared with Mixed-race [13.0% (95%CI 12.7–13.4)], Black [13.2% (95%CI 12.5–13.8)], and White [13.5% (95%CI 13.1–13.8)] populations after adjustment. Indigenous peoples showed the highest average consumption of this food group but with a wide confidence interval [13.4% (95%CI 10.8–16.0)].

After adjustment, the consumption of ultra-processed foods decreased by approximately 2 pp for the two categories of race/skin color with the highest averages: White [18.6% (95%CI 18.2–19.1)] and Yellow [22.4% (95%CI 18.6–26.2)] individuals. On the other hand, the average consumption of Black [17.7% (95%CI 16.8–18.6)] and Mixed-race [17.8% (95%CI 17.3–18.3)] people increased by approximately 1 pp. The adjustment increased the energy contribution of this food group in the diet of Indigenous peoples [18.6% (95%CI 15.6–21.6)], which decreased the differences from the other groups.

The crude and adjusted estimates for each of the 31 subgroups are presented in the following tables. The main results of the analyses after adjustment are described below. In general, for most food items and culinary preparations, the Yellow and indigenous populations showed no difference in consumption compared with the other categories.

Table 2 shows that in the group of *in natura* and minimally processed foods, rice had the greatest energy contribution for all categories of race/skin color; however, consumption was higher among Black [12.0% (95%CI 11.5–12.5)] and Mixed-race [11.8% (95%CI 11.6–12.1)] populations, when compared with Whites [11.3% (95%CI 11.0–11.5)]. The other groups showed no statistical difference, although Yellow individuals [12.7% (95%CI 10.7–14.7)] presented the highest mean for the participation of this item in the diet.

**Table 2:** Average daily energy contribution (% kcal/day) of *in natura* and minimally processed foods, according to race/skin color. Brazil, 2017–2018.

Race/skin color	Crude	Adjusted^a^	Crude	Adjusted^a^	Crude	Adjusted^a^	Crude	Adjusted^a^
	**Rice**		**Beef and pork**		**Mixed preparations^b^**		**Beans**	
Mixed-race	12.3 (12.1–12.6)	11.8 (11.6–12.1)	10.1 (9.9–10.4)	10.2 (10.0–10.4)	8.2 (7.9–8.5)	8.4 (8.1–8.7)	8.0 (7.8–8.2)	7.8 (7.6–8.0)
White	10.6 (10.4–10.9)	11.3 (11.0–11.5)	10.2 (10.0–10.5)	10.1 (9.9–10.4)	8.6 (8.3–8.9)	8.4 (8.1–8.8)	6.6 (6.4–6.8)	7.0 (6.8–7.2)
Black	12.6 (12.1–13.1)	12.0 (11.5–12.5)	10.3 (9.8–10.8)	10.4 (9.9–10.9)	8.3 (7.6–9.0)	8.4 (7.8–9.0)	8.5 (8.1–8.9)	8.0 (7.6–8.4)
Yellow	11.6 (9.6–13.5)	12.7 (10.7–14.7)	8.8 (7.1–10.5)	9.1 (7.5–10.6)	10.5 (7.9–13.0)	10.1 (7.6–12.6)	5.6 (4.6–6.6)	6.6 (5.6–7.5)
Indigenous	11.4 (9.8–13.0)	11.3 (9.7–12.9)	10.4 (8.6–12.1)	10.6 (8.8–12.4)	7.6 (5.7–9.5)	7.5 (5.7–9.4)	6.7 (4.3–9.0)	6.6 (4.1–9.2)
	**Poultry meats**		**Fruits and 100% fruit juice**		**Homemade desserts^c^**		**Coffees and teas**	
Mixed-race	6.8 (6.6–6.9)	6.5 (6.4–6.7)	4.4 (4.3–4.5)	4.7 (4.6–4.9)	3.0 (2.9–3.1)	3.1 (3.0–3.2)	2.3 (2.2–2.4)	2.4 (2.3–2.5)
White	5.9 (5.8–6.0)	6.2 (6.0–6.3)	5.2 (5.0–5.3)	4.8 (4.7–4.9)	3.4 (3.2–3.6)	3.2 (3.1–3.4)	2.6 (2.5–2.7)	2.5 (2.4–2.6)
Black	6.4 (6.2–6.7)	6.3 (6.0–6.5)	4.6 (4.3–4.9)	4.8 (4.6–5.1)	2.9 (2.7–3.2)	3.1 (2.8–3.3)	2.4 (2.3–2.6)	2.4 (2.3–2.6)
Yellow	5.6 (4.7–6.6)	6.1 (5.2–7.0)	5.8 (4.5–7.2)	5.1 (3.8–6.4)	2.4 (1.6–3.2)	2.3 (1.4–3.1)	1.8 (1.3–2.3)	1.8 (1.2–2.3)
Indigenous	6.6 (5.0–8.1)	6.4 (4.9–8.0)	4.2 (3.2–5.2)	4.3 (3.4–5.1)	3.4 (2.2–4.6)	3.5 (2.3–4.7)	2.3 (1.7–2.8)	2.2 (1.7–2.8)
	**Tubers and roots**		**Pasta**		**Vegetables**		**Eggs**	
Mixed-race	2.0 (1.9–2.1)	2.1 (2.0–2.2)	2.1 (2.0–2.2)	2.1 (2.0–2.1)	1.2 (1.2–1.3)	1.4 (1.3–1.4)	1.6 (1.5–1.7)	1.5 (1.5–1.6)
White	2.2 (2.1–2.3)	2.1 (2.0–2.2)	2.0 (1.9–2.2)	2.0 (1.9–2.2)	1.7 (1.6–1.7)	1.5 (1.4–1.5)	1.5 (1.4–1.6)	1.6 (1.5–1.7)
Black	2.1 (1.9–2.3)	2.2 (2.0–2.4)	2.1 (1.9–2.3)	2.1 (1.9–2.3)	1.3 (1.2–1.4)	1.4 (1.3–1.5)	1.5 (1.3–1.6)	1.4 (1.3–1.5)
Yellow	1.7 (1.2–2.1)	1.6 (1.1–2.0)	1.8 (1.1–2.5)	2.0 (1.3–2.7)	2.0 (1.5–2.5)	1.7 (1.2–2.2)	1.7 (0.7–2.7)	1.8 (0.7–2.8)
Indigenous	2.9 (0.9–5.0)	3.0 (0.9–5.1)	2.0 (1.4–2.6)	2.0 (1.4–2.6)	1.3 (0.9–1.7)	1.4 (1.0–1.8)	1.3 (0.9–1.7)	1.3 (0.9–1.7)
	**Fish**		**Corn, oats, and wheat (excluding flours)**		**Cassava flour**		**Other^d^**	
Mixed-race	1.7 (1.6–1.8)	1.4 (1.3–1.5)	1.5 (1.4–1.5)	1.2 (1.1–1.3)	1.9 (1.8–2.0)	1.4 (1.3–1.5)	3.1 (2.9–3.3)	3.0 (2.8–3.1)
White	1.0 (0.9–1.1)	1.3 (1.2–1.4)	0.9 (0.8–1.0)	1.2 (1.1–1.3)	0.8 (0.8–0.9)	1.3 (1.2–1.4)	3.2 (3.0–3.4)	3.3 (3.1–3.5)
Black	1.3 (1.1–1.5)	1.3 (1.1–1.4)	1.3 (1.1–1.5)	1.2 (1.0–1.3)	1.6 (1.4–1.8)	1.5 (1.3–1.7)	2.6 (2.4–2.9)	2.7 (2.5–3.0)
Yellow	1.3 (0.4–2.1)	1.4 (0.6–2.2)	0.6 (0.3–0.8)	1.1 (0.8–1.3)	1.4 (0.8–2.1)	1.7 (1.0–2.4)	3.5 (2.2–4.8)	3.2 (1.9–4.5)
Indigenous	2.4 (1.2–3.5)	2.2 (1.3–3.2)	1.0 (0.5–1.4)	0.9 (0.5–1.3)	1.9 (0.9–2.8)	1.6 (1.0–2.1)	3.4 (1.8–4.9)	3.2 (1.9–4.5)

^a^ Adjustment for gender, age, income, schooling, area of residence, and macro-region of the country.

^b^ Mixed preparations including rice, meat of any origin, and other vegetables.

^c^ Includes cakes, breads, pies, and other sweet desserts such as simple cakes, ambrosia, coconut candy, Brazilian corn pudding, Brazilian rice pudding, caramelized banana, condensed milk dessert with cashews, Brazilian coconut egg custard, milk pudding, Brazilian prune coconut candy, pineapple delight, carrot cake with chocolate filling.

^d^ Includes seafood, other meats, natural yogurt, other flours, milk, offal, breads and salted pies, other legumes, nuts, fungi, olive oil and other oils, butter, sugar, lard, coconut milk, cream, and other culinary ingredients.

A similar pattern was observed for the consumption of beans, whose energy contribution was higher among Black [8.0% (95%CI 7.6–8.4)] and Mixed-race [7.8% (95%CI 7.6–8.0)] populations, compared with the White population [7.0% (95%CI 6.8–7.2)]. Indigenous [6.6% (95%CI 4.1–9.2)] and Yellow [6.6% (95%CI 5.6–7.5)] people had a similar consumption, lower than the other groups.

Regarding poultry, Mixed-race individuals [6.5% (95%CI 6.4–6.7)] showed higher consumption than Whites [6.2% (95%CI 6.0–6.3)] and the other categories showed no significant difference.

The other items in the group showed no statistical differences between race/skin color categories, but mixed preparations that combine rice, meat, and vegetables had a higher contribution for Yellow individuals (10.1%) compared with the others, and the Indigenous individuals were those with lower consumption of these foods (7.5%). On the other hand, the indigenous were those with the highest absolute consumption of fish (2.2%) and roots and tubers (3.0%). Yellow (1.7%) and White (1.5%) individuals had a slightly higher consumption of vegetables and leafy greens compared to the other groups.

Regarding the subgroups of processed foods, the preparations with the greatest energy contribution for all categories of race/skin color were non-ultraprocessed bread and sandwiches (also made with non-ultraprocessed bread), as shown in Table 3. The consumption of this food group showed the lowest energy contribution to the Yellow population [7.5% (95%CI 6.3–8.7)], with a statistical difference from the others, and higher absolute consumption by the Indigenous population [11.2% (95%CI 8.8–13.7)]. The consumption of White, Mixed-race, and Black individuals showed no differences, with means between 10.4% and 10.6%. The White population had a consumption of processed cheeses [0.9% (95%CI 0.8–1.0)] with significant differences from the Black [0.6% (95% CI 0.5–0.7)] and Mixed-race [0.7% (95%CI 0.6–0.7)] populations.

**Table 3:** Average daily energy contribution (% kcal/day) of processed foods, according to race/skin color. Brazil, 2017–2018.

Race/skin color	Crude	Adjusted^a^	Crude	Adjusted^a^	Crude	Adjusted^a^	Crude	Adjusted^a^
	**Non-ultraprocessed bread and sandwiches^b^**		**Beers and wines**		**Cheeses**		**Salted/dried/smoked/cured meats**	
Mixed-race	10.7 (10.4–10.9)	10.4 (10.1–10.7)	1.0 (0.9–1.2)	1.2 (1.0–1.4)	0.5 (0.5–0.6)	0.7 (0.6–0.7)	0.5 (0.5–0.6)	0.4 (0.3–0.5)
White	10.2 (9.9–10.5)	10.6 (10.3–10.9)	1.3 (1.2–1.4)	1.1 (1.0–1.2)	1.1 (1.0–1.2)	0.9 (0.8–1.0)	0.3 (0.2–0.3)	0.4 (0.4–0.5)
Black	11.1 (10.5–11.8)	10.6 (10.0–11.2)	1.0 (0.9–1.2)	1.2 (1.0–1.3)	0.5 (0.4–0.6)	0.6 (0.5–0.7)	0.6 (0.4–0.8)	0.5 (0.4–0.7)
Yellow	6.9 (5.6–8.2)	7.5 (6.3–8.7)	0.6 (0.2–1.1)	0.3 (0.0–0.8)	1.3 (0.0–2.7)	1.0 (0.0–2.3)	0.3 (0.0–0.5)	0.4 (0.2–0.6)
Indigenous	11.5 (8.8–14.3)	11.2 (8.8–13.7)	0.8 (0.2–1.4)	0.9 (0.3–1.5)	0.9 (0.2–1.5)	0.9 (0.3–1.5)	0.3 (0.0–0.5)	0.2 (0.0–0.4)
	**Other^c^**							
Mixed-race	0.4 (0.3–0.4)	0.4 (0.3–0.4)						
White	0.4 (0.4–0.5)	0.4 (0.3–0.4)						
Black	0.3 (0.2–0.3)	0.3 (0.2-0.4)						
Yellow	0.2 (0.0–0.3)	0.1 (0.0–0.3)						
Indigenous	0.2 (0.0–0.4)	0.2 (0.0–0.4)						

^a^ Adjustment for gender, age, income, schooling, area of residence, and macro-region of the country.

^b^ Includes only non-ultraprocessed bread (“*pão francês*”) sandwiches.

^c^ Vegetable preserves, fruit jam, processed nuts, legume preserves, tomato sauce, fish preserve.

The consumption of beers and wines was lower for the Yellow population [0.3% (95%CI 0.0–0.8)] – significantly lower than for the others – and for Indigenous population [0.9% (95%CI 0.3–1.5)]. The other items that make up the group of processed foods showed no significant differences between the categories of race/skin color.

In the ultra-processed foods group, despite the absence of significant differences between the categories of race/skin color for the items after the adjustment of the analyses, the foods with the highest energy contribution were the ready-to-eat snacks, which includes fast food sandwiches, fried and baked snacks, etc., as shown in Table 4. Despite the absence of statistical significance in comparison to the other categories, Yellow individuals showed the highest average consumption of this food group, which contributed with 6.8% (95%CI 4.9–8.6) of energy, whereas Indigenous individuals had the lowest consumption, with 3.7% (95%CI 2.3–5.1). The other categories are intermediate in consumption and the White population [5.8% (95%CI 5.5–6.1)] consumed slightly more ultra-processed foods than Black [5.3% (95%CI 4.5–6.0)] and Mixed-race [5.4% (95%CI 4.9–5.8)] populations.

**Table 4:** Average daily energy contribution (% kcal/day) of ultra-processed foods, according to race/skin color. Brazil, 2017–2018.

Race/skin color	Crude	Adjusted^a^	Crude	Adjusted^a^	Crude	Adjusted^a^	Crude	Adjusted^a^
	**Bread, snacks, and fast food dishes^b^**		**Crackers and chips**		**Sweet cookies and baked goods**		**Soft drinks**	
Mixed-race	4.6 (4.1–5.0)	5.4 (4.9–5.8)	2.8 (2.7–2.9)	2.7 (2.5–2.8)	2.2 (2.1–2.3)	2.2 (2.1–2.3)	1.4 (1.4–1.5)	1.5 (1.4–1.6)
White	6.8 (6.4–7.1)	5.8 (5.5–6.1)	2.6 (2.4–2.7)	2.7 (2.6–2.8)	2.2 (2.1–2.4)	2.2 (2.1–2.3)	1.6 (1.5–1.6)	1.4 (1.4–1.5)
Black	4.7 (3.9–5.4)	5.3 (4.5–6.0)	2.8 (2.5–3.0)	2.8 (2.5–3.0)	2.0 (1.8–2.2)	2.1 (1.8–2.3)	1.4 (1.2–1.5)	1.5 (1.3–1.6)
Yellow	8.3 (6.4–10.2)	6.8 (4.9–8.6)	2.6 (1.7–3.6)	2.7 (1.8–3.6)	2.0 (1.2–2.8)	2.2 (1.5–2.9)	1.9 (1.3–2.6)	1.9 (1.3–2.6)
Indigenous	3.2 (1.4–3.4)	3.7 (2.3–5.1)	3.0 (1.9–4.0)	2.9 (1.9–3.9)	2.9 (1.5–4.3)	3.0 (1.5–4.4)	2.1 (1.1–3.1)	2.2 (1.3–3.0)
	**Sweets^c^**		**Sausages**		**Yogurts and dairy drinks**		**Ready or semi-ready dishes^d^**	
Mixed-race	0.9 (0.9–1.0)	1.1 (1.0–1.2)	1.3 (1.2–1.4)	1.3 (1.2–1.4)	0.9 (0.8–1.0)	1.0 (0.9–1.1)	0.9 (0.8–1.0)	0.9 (0.8–1.1)
White	1.4 (1.3–1.5)	1.2 (1.1–1.4)	1.3 (1.2–1.4)	1.3 (1.2–1.4)	1.3 (1.2–1.4)	1.1 (1.0–1.2)	1.2 (1.0–1.3)	1.1 (1.0–1.2)
Black	1.1 (0.9–1.3)	1.2 (1.1–1.4)	1.3 (1.2–1.4)	1.3 (1.1–1.4)	0.8 (0.7–1.0)	0.9 (0.8–1.1)	0.9 (0.6–1.1)	0.9 (0.7–1.2)
Yellow	1.9 (0.6–3.2)	1.6 (0.3–2.8)	1.7 (0.9–2.5)	1.8 (1.0–2.5)	1.0 (0.5–1.5)	0.8 (0.2–1.3)	0.6 (0.0–1.2)	0.5 (0.0–1.1)
Indigenous	0.9 (0.3–1.5)	1.0 (0.3–1.6)	1.4 (0.7–2.2)	1.4 (0.8–2.1)	1.8 (0.1–3.5)	1.9 (0.3–3.4)	0.8 (0.2–1.3)	0.8 (0.2–1.4)
	**Artificial juice and other non-alcoholic beverages**		**Other^e^**					
Mixed-race	1.2 (1.1–1.2)	1.2 (1.1–1.3)	0.5 (0.4–0.5)	0.5 (0.5–0.6)				
White	1.2 (1.1–1.3)	1.2 (1.1–1.3)	0.7 (0.6–0.8)	0.6 (0.5–0.7)				
Black	1.3 (1.2–1.5)	1.4 (1.2–1.5)	0.4 (0.3–0.5)	0.5 (0.4–0.6)				
Yellow	3.3 (0.0–6.7)	3.2 (0.0–6.5)	1.4 (0.4–2.4)	1.3 (0.4–2.3)				
Indigenous	1.0 (0.3–1.8)	1.0 (0.3–1.8)	0.7 (0.0–1.4)	0.7 (0.0–1.5)				

^a^ Adjustment for gender, age, income, schooling, area of residence, and macro-region of the country.

^b^ Includes burgers, hot dogs, pizzas, fried and baked snacks, and the like.

^c^ It includes candies, confectionery, chocolates, ice cream, and the like.

^d^ Frozen pasta or meat dishes, instant noodles, and powdered soups.

^e^ Includes heavy cream, processed curd, cream cheese, margarine, breakfast cereals, distilled alcoholic beverages and drinks with these beverages, industrialized sauces, and supplements.

For the Yellow population, the highest specific estimates of consumption of ultra-processed snacks and bread (6.8%), artificial juice and other non-alcoholic beverages (3.2%), sausages (1.8%), and sweets (1.6%) stand out, whereas Indigenous people consume fewer energy from ultra-processed bread (3.7%) but have a slightly higher percentage of consumption of soft drinks (1.9%) and dairy beverages (1.9%) compared with the other categories.

## DISCUSSION

Our results reveal that the Black and Mixed-race populations corresponded to the category with the higher consumption of *in natura* and minimally processed foods, whereas the White and Yellow populations consumed more ultra-processed foods when compared with the others. Yellow individuals also showed the lowest consumption of processed foods.

Current dietary recommendations, including the Dietary Guidelines for the Brazilian Population, indicate that a healthy diet should be based on *in natura* and minimally processed foods, with a limited amount of processed foods, and avoiding the consumption of ultra-processed foods^14,24^. Although the Mixed-race, Black, and Indigenous categories of race/skin color presented a consumption converging to the recommended, the changes observed in the analyses with and without adjustment for confounding variables indicate that these differences may be associated with socioeconomic and demographic conditions. Food intake is the result of the interaction of different factors, and is influenced by cultural characteristics, availability, and access to food. In our analyses, part of the variations between the categories of race/skin color was explained by socioeconomic and demographic characteristics, demonstrated by reducing the magnitude of the differences in the adjusted models.

Regarding the *in natura* and minimally processed foods, in the crude analyses, White and Yellow groups showed higher averages of consumption of fruits, leafy greens, and vegetables, a difference that did not remain after adjustment. On the other hand, the higher differences that remained after adjustment were observed in the averages of rice and beans consumption, items for which the Mixed-race and Black categories presented the highest values, with statistical differences from the others. Although our results indicate that Blacks and Mixed-race people presented greater convergence to the recommendations of the Dietary Guidelines for the Brazilian Population regarding the greater relative participation of rice and beans preparations, this does not represent a higher overall quality of food, since other foods such as fruits, leafy greens, and vegetables lacked the same association. Other studies that included food intake in the analyses also found that Black and Mixed-race people have lower recommended or regular consumption of fruits and vegetables and higher consumption of beans^7,10,11,13^. The absence of statistical significance was particularly important for ultra-processed foods, a group in which none of the differences observed in the crude analyses remained after adjustment.

The literature is consistent in concluding that income is an important factor associated with food consumption, which in turn is related to access influenced by food prices^25^. Since the 1990s, ultra-processed foods prices showed a decreasing trend in Brazil, although still being more expensive than *in natura*, minimally processed and processed foods. The average lower price of grains such as rice and beans can contribute to making the traditional Brazilian dishes more accessible^28^.

Given this evidence, interpreting the results on food consumption needs to be done in the light of the social processes that occurred in Brazil, a country with a history of slavery, whose consequences are marked by inequalities directly linked to ethnic-racial issues^29^. According to official IBGE data, Black and Mixed-race people show higher proportions of illiterate individuals working in informality and lower average income, compared with the White population^30^. Similarly, Indigenous peoples suffer from marginalization, poverty, and discrimination^31,32^. In addition, data from the POF 2017–2018 also indicated that the Black and Mixed-race groups show a higher frequency of food insecurity^33^, a result suggesting that, even with higher relative consumption of rice and beans compared with more socioeconomically privileged groups, the diet of the Black and Mixed-race population reflect the condition of social vulnerability to which they are subjected. Thus, the inequalities observed in food consumption can be attributed not only to the characteristics of racial groups, but to their social, economic, and demographic conditions.

Due to the complexity of racial relations in Brazil, we chose to use in this study the five categories of race or skin color defined by the IBGE to collect data from the Brazilian population, since skin color in the country is an important social marker of inequalities^34^. In addition, the use of the five categories shows possible inequities to more vulnerable racial groups.

Despite being a nationally-representative survey, the POF is not designed to be representative of the groups defined by race or skin color and, thus, the sample size for some categories – specifically Yellow and Indigenous – does not contemplate population groups equitably, and does not present sufficient statistical power to identify possible differences, which is a constant limitation for evaluating the Brazilian population.

In addition to this limiting characteristic of sampling surveys, many studies choose not to include the smaller groups in the analyses^8,10,13^. Some authors used a specific category (*Negros,* in Brazilian Portuguese), created from the combination of Black and Mixed-race groups, as a single stratum of analysis, and others used a binary approach, comparing two large groups defined as *White* and *non-White,* treating the former as normative and hiding the differences between the other categories of race/skin color^9,12^.

Although, on the one hand, consistent similarities appeared in the food intake of Black and Mixed-race populations and, on the other hand, some items showed no statistical differences, due to the wide confidence intervals for Indigenous and Yellow individuals, describing some important differences was possible. The higher consumption of rice and mixed preparations of rice, meat, and legumes for the Yellow population and fish, roots and tubers for Indigenous peoples are examples that illustrate cultural markers of food.

Our results show the marked presence of rice and beans in the Brazilian diet. Present throughout the country, it is not known exactly when this combination came to the Brazilians’ table, but from the 20th century onward its presence is seen in everyday life and typical dishes of the different regions. Also, beans, also known as “poor’s meat,” with rice have relevant importance mainly in homes with lower purchasing power in the country, considered healthy for its nutritional combination^35^.

It is worth mentioning that the differences observed may vary in magnitude when considering the geographical distribution of groups across the country and, thus, future analyses should also explore subnational inequalities.

Population surveys and health information systems have included the race/skin color field in their forms, but, in addition to often being left blank^3,36^, incorporating the data collected in analyses, as a fundamental part of the process of understanding the epidemiological situation of the Brazilian population, is still necessary. Studies on inequalities in behaviors and prevalence of risk factors by population race/skin color are scarce and, often, this information appears diluted in analyses that include other social dimensions.

Some methodological limitations are common to surveys that collect food consumption data, including possible bias in the measurement of the usual diet, participant recall bias, and lack of precision in consumption measures. To reduce their impacts on the results, validated questionnaires were used and the collection had quality control, among other standard procedures of data manipulation^17,37^.

## CONCLUSIONS

This study is an important step towards filling a gap in the scientific literature on the variation of food intake among ethnoracial groups in Brazil. Differences were found in the participation of *in natura* and minimally processed foods and ultra-processed foods in the diet of the Brazilian population, which appear associated with the socioeconomic position of individuals in society that, in general, are unfavorable for Black, Mixed-race, and Indigenous people. Therefore, initiatives and public policies to reduce inequalities that disproportionately affect racialized groups need to occur concomitantly with those aimed at encouraging the consumption of *in natura* and minimally processed foods and reducing the consumption of ultra-processed foods, whose participation has grown in the diet of the Brazilian population, with harmful effects to everybody’s health. Given this evidence, continued efforts should be made for more studies to include the epidemiological description of the population by race/skin color, contributing to public health and understanding and tackling inequities.
