# Supplementary material for: Differences in food consumption of the Brazilian population by race/skin color in 2017–2018
Source: Rev Saude Publica. 2023 Feb 8;57:4. doi: 10.11606/s1518-8787.2023057004000 (PMC9933641; doi:10.11606/s1518-8787.2023057004000)
Supplement: Translation

# Translation: Diferenças no consumo alimentar da população brasileira por raça/cor da pele em 2017–2018

**Authors:** Janaína Calu Costa, Amanda Cristina da Silva de Jesus, Juliana Giaj Levra de Jesus, Mariana Ferreira Madruga, Thays Nascimento Souza, Maria Laura da Costa Louzada

**Affiliations:** I Brasil Universidade Federal de Pelotas. Centro Internacional para Equidade em Saúde. Programa de Pós-Graduação em Epidemiologia. Pelotas, RS, Brasil; II Brasil Universidade de São Paulo. Faculdade de Saúde Pública. Programa de Pós-Graduação em Nutrição em Saúde Pública. São Paulo, SP, Brasil; III Brasil Universidade de São Paulo. Faculdade de Medicina. Programa de Pós-Graduação em Saúde Coletiva. São Paulo, SP, Brasil.; IV Brasil Universidade de São Paulo. Núcleo de Pesquisas Epidemiológicas em Nutrição e Saúde. São Paulo, SP, Brasil

**Keywords:** Alimentos, Dieta e Nutrição, Fatores Raciais, Fatores Socioeconômicos, Inquéritos Nutricionais

## Abstract

**OBJETIVO:**

Avaliar o consumo alimentar no Brasil por raça/cor da pele da população.

**MÉTODOS:**

Foram analisados dados de consumo alimentar da Pesquisa de Orçamentos Familiares 2017–2018. Alimentos e preparações culinárias foram agrupados em 31 itens, compondo três grupos principais, definidos por características do processamento industrial: 1 – *in natura*/minimamente processados, 2 – processados e 3 – ultraprocessados. O percentual de calorias de cada grupo foi estimado por categorias de raça/cor da pele – branca, preta, parda, indígena e amarela –, utilizando-se regressão linear bruta e ajustada para sexo, idade, escolaridade, renda, macrorregião e área.

**RESULTADOS:**

Nas análises brutas, o consumo de alimentos *in natura*/minimamente processados foi menor para amarelos [66,0% (Intervalo de Confiança 95% 62,4–69,6)] e brancos [66,6% (IC95% 66,1–67,1)] que para pretos [69,8% (IC95% 68,9–70,8)] e pardos [70,2% (IC95% 69,7–70,7)]. Amarelos consumiram menos alimentos processados, com 9,2% das calorias (IC95% 7,2–11,1) enquanto os demais consumiram aproximadamente 13%. Ultraprocessados foram menos consumidos por pretos [16,6% (IC95% 15,6–17,6)] e pardos [16,6% (IC95% 16,2–17,1)], e o maior consumo ocorreu entre brancos [20,1% (IC95% 19,6–20,6)] e amarelos [24,5% (IC95% 20,0–29,1)]. O ajuste dos modelos reduziu a magnitude das diferenças entre as categorias de raça/cor da pele. A diferença entre pretos e pardos em relação aos brancos diminuiu, de 3 pontos percentuais (pp), para 1,2 pp no consumo de alimentos *in natura*/minimamente processados e as maiores diferenças remanescentes foram no consumo de arroz e feijão, com maior percentual na alimentação de pretos e pardos. A participação de alimentos processados permaneceu aproximadamente 4 pp menor para amarelos. O consumo de ultraprocessados diminuiu aproximadamente 2 pp para brancos e amarelos; por outro lado, aumentou 1 pp no consumo de pretos, pardos e indígenas.

**CONCLUSÃO:**

Diferenças no consumo alimentar segundo raça/cor da pele foram encontradas e são influenciadas por condições socioeconômicas e demográficas.

## INTRODUÇÃO

A população brasileira é caracterizada por uma grande diversidade étnico-racial, o que se reflete na cultura e na identidade do país^1^. Essa diversidade, porém, está associada a importantes iniquidades nas condições de vida e saúde da população, visto que alguns grupos se encontram em situação de maior vulnerabilidade, devido aos processos sócio-históricos que contribuíram para sua segregação e marginalização, especialmente aqueles identificados racialmente, como a população preta, parda e indígena^2,3^.

As situações de desvantagem econômica e social em que esses grupos se encontram são reflexos dos mecanismos pelos quais o racismo contribui para as desigualdades raciais na saúde, estabelecendo-o como um importante determinante social da saúde^4,5^. Desse modo, o racismo influencia de maneira significativa sobre as condições de sobrevivência, o acesso a serviços, e os comportamentos, incluindo aqueles relacionados à alimentação.

Assim como estimativas nacionais mascaram desigualdades subnacionais, analisar a saúde da população por outras dimensões sociais não é suficiente para a identificação das desigualdades raciais. O uso da informação sobre raça/cor da pele é uma forma de dar visibilidade estatística aos grupos e possibilitar a tomada de medidas que atendam às diferentes demandas. Apesar da coleta habitual da informação de cor da pele ou raça em inquéritos populacionais oficiais e da obrigatoriedade do campo em formulários e sistemas de informação em saúde no Brasil, poucos estudos sobre consumo alimentar incluem análises de desigualdades por categorias de raça/cor da pele^6^.

Recomendações do Guia Alimentar para a População Brasileira e os princípios orientadores de seu conteúdo incluem a compreensão de que a alimentação é mais do que a ingestão de nutrientes, refletindo contextos sociais, padrões e tradições alimentares e o impacto da produção e do consumo^14^. As recomendações são baseadas na classificação NOVA, que agrupa os alimentos em categorias de acordo com a extensão e o propósito do processamento industrial, e que tem se mostrado útil para a compreensão da epidemiologia de doenças e dos impactos do consumo nos sistemas alimentares em todo o mundo^14-16^.

As tendências no Brasil mostram um aumento do consumo de alimentos ultraprocessados e concomitante declínio do consumo de alimentos *in natura* e minimamente processados^17,18^. As estimativas, entretanto, costumam ser apresentadas estratificadas por sexo, idade, região, área e faixas de renda, mas não por categorias de raça ou cor da população, invisibilizando possíveis desigualdades entre esses grupos^17,18^.

A fim de contribuir com o preenchimento dessa lacuna, o objetivo do estudo foi avaliar o consumo alimentar no Brasil em 2017–2018, segundo características do processamento industrial e de acordo com a raça/cor da pele da população.

## MÉTODOS

### Fonte de Dados e Amostragem

Os dados analisados compõem o módulo de consumo alimentar pessoal da Pesquisa de Orçamentos Familiares (POF), realizada pelo Instituto Brasileiro de Geografia e Estatística (IBGE) entre julho de 2017 e julho de 2018^17^. Um processo de amostragem complexa em dois estágios foi realizado, com agrupamento de setores censitários com estratificação geográfica e socioeconômica e posterior sorteio deles no primeiro estágio, além de sorteio de domicílios pertencentes aos setores selecionados no segundo estágio^17^.

### Consumo Alimentar

As informações referentes ao consumo alimentar individual foram coletadas em uma subamostra de 20.112 domicílios e reportadas por moradores com 10 anos ou mais de idade^17^. Aos 46.164 indivíduos selecionados para o módulo de consumo, foram aplicados inquéritos recordatórios de 24 horas em dois dias não consecutivos^17^.

A quantidade de cada alimento ou bebida registrada nos recordatórios foi transformada em gramas ou mililitros e convertidas em energia (quilocalorias, kcal) com base na Tabela Brasileira de Composição de Alimentos^19^. Alimentos e preparações culinárias foram agrupados com base na classificação NOVA, que classifica os itens alimentares de acordo com características do processamento industrial^20^. A categorização foi adaptada, segundo metodologia utilizada por Louzada et al. (2015), em que não há a decomposição das preparações em ingredientes, prevalecendo as características dos itens principais reportados^21^. Os três grupos principais são compostos por 31 subgrupos criados a partir dos alimentos consumidos de forma isolada ou em preparações culinárias com múltiplos ingredientes, conforme descrito a seguir e detalhado no Quadro.

**Quadro:** Classificação dos alimentos relatados na Pesquisa de Orçamento Familiar 2017–2018 segundo grupos e subgrupos da classificação de alimentos NOVA.

Grupos da NOVA		Alimentos
Alimentos in natura ou minimamente processados, ingredientes culinários e preparações culinárias		
1	Arroz	Arroz branco, parboilizado, agulha, integral, 3 grãos e 7 grãos Preparações feitas à base de arroz (bolinho de arroz, arroz de coco, arroz de leite, risoto)
2	Carnes de boi e de porco	Carne bovina ou suína (bife, filé mignon, músculo, costela bovina, carne moída, costela suína, pernil, lombo)
		Preparações com carnes vermelhas (kafta, carne bovina desfiada, rosbife, pernil assado, bisteca grelhada, leitão a pururuca)
3	Feijão	Feijão preto, carioca, verde, mulatinho roxo, corda, andu
		Preparações à base de feijão (sopa de feijão e caldo de feijão)
4	Carne de aves	Carne de frango, peru ou pato
		Preparações com aves (frango a passarinho, peru ou pato assado, frango caipira, filé de frango grelhado)
5	Frutas e suco de frutas natural	Frutas cruas ou cozidas, salada de frutas
		Suco de frutas natural
6	Macarrão	Variedade de tipos de macarrão, nhoque, panqueca
		Preparações com macarrão (Macarrão com queijo, macarrão alho e óleo, macarrão com molho branco, macarrão gratinado)
7	Verduras e legumes	Legumes e verduras crus, refogados, fritos, assados ou cozidos
		Preparações de legumes e verduras (vinagrete, saladas e sopas variadas, quibebe, caponata, jardineira de legumes, creme de espinafre, quibe vegetariano, ratatouille)
8	Tubérculos e raízes	Batata inglesa ou doce, inhame, cará, mandioca ou aipim ou macaxeira, mandioquinha
		Preparações como purês
9	Ovos	Ovos de galinha e codorna
		Preparações com ovos (ovos mexidos, ovos moles, omelete, suflê, gemada, macarrão com ovos)
10	Farinha de mandioca	Farinha de mandioca, farinha d’água, tapioca
		Preparações de farinha de mandioca (farofa simples, cruera, sagu de tapioca)
11	Milho, aveia e trigo (excluindo farinhas)	Quinoa, trigo, milho
		Preparações como cuscuz, creme de milho, polenta, sopa de fubá
12	Peixes	Peixes de água salgada ou doce
		Preparações com peixes (sashimi, peixe com camarão, peixe gratinado, peixada, tartare de peixe)
13	Cafés e Chás	Chás feitos de ervas variadas (camomila, capim limão, preto, etc.)), mate, chimarrão, tererê
		Cafés (expresso, carioca, com leite, descafeinado)
14	Preparações mistas de arroz/ macarrão/ farinhas ou outros cereais + carnes de boi/ porco/ aves ou peixes/ frutos do mar + tubérculos/ feijão/ legume / verduras ou preparações com alimentos in natura ou minimamente processados^a^	Bife a rolê, carne de panela, feijoada, costela com legumes, carne de sol com macaxeira, carne suína com legumes
		Moquecas variadas, bobo de camarão, acarajé, peixe assado com legumes, temaki
		Bife à milanesa, hambúrguer, macarrão com carne, paçoca de carne de sol
		Arroz com feijão, legumes à milanesa, arroz a grega, cuscuz paulista, tabule, lasanha de berinjela
		Galinhada, arroz ao molho pardo, frango à parmegiana
		Cuscuz de atum, pirão, risoto de camarão, arroz com frutos do mar
		Arroz de cuxa, lasanha de frutos do mar
		Baião de dois, arrumadinho, bife a rolê, angu com carne moída e tomate
		Salpicão, galinhada com pequi, charuto de repolho com frango
		Strogonoff, salada de maionese, açaí com granola, arroz ou macarrão com linguiça ou salsicha, leguminosas com linguiça, camarão com requeijão cremoso
15	Sobremesas caseiras	Bolos, pães, tortas e outras sobremesas doces (Bolos simples, ambrosia, cocada, curau, arroz doce, banana caramelizada, cajuzinho, quindim)
		Bolos e tortas doces com alimentos in natura ou minimamente processados e ultraprocessados (pudim de leite, olho de sogra, delícia de abacaxi, bolo de cenoura com recheio de chocolate)
16	Outros	Frutos do mar (camarão, polvo, lula, mariscos, ovas)
		Outras carnes (carne de bode, cabrito, carneiro, paca, jacaré, capivara, jabuti, cordeiro e outros animais)
		Iogurte natural (kefir, coalhada, iogurte natural e iogurte desnatado)
		Outras farinhas (farinha de aveia, copioba ou milho, mingau, guaraná em pó, vatapá, cuscuz marroquino)
		Miúdos (vísceras de boi, frango, bode, porco) e preparações como sarapatel, dobradinha ensopada, sarrabulho
		Leite (vaca ou cabra, integral, semidesnatado ou desnatado, em pó ou líquido, com ou sem lactose), nata
		Pães e tortas salgadas (pão caseiro, quiche, tortas variadas)
		Outras leguminosas (ervilha, fava, grão de bico, lentilha, soja em grão, carne e proteína de soja)
		Nozes e sementes (inclui amendoim): Castanhas, amêndoa, avelã, amendoim, pupunha, buriti, gergelim, linhaça
		Fungos (cogumelos in natura)
		Açúcares, óleos (azeite, óleo de soja, milho, coco), manteiga, banha, leite de coco, vinagre e sal
		Fécula (tapiocas recheadas, cuscuz de tapioca, sagu de vinho tinto)
		Água
**Alimentos processados**		
17	Pão francês e sanduíches	Pão francês ou pão d’água ou pão de trigo ou cacetinho (simples ou integral), *bruschetta*
		Pão com manteiga ou sanduíche de pães processados e com recheios de alimentos in natura ou minimamente processados ou alimentos processados (pão com rosbife, pão com sardinha, pão com queijo, pão com ovo, pão com frango ou carne)
18	Queijos	Queijo prato, queijo mussarela, ricota, gorgonzola, coalho
19	Carnes salgadas/ secas/ defumadas/ curadas	Carne seca, carne de sol, bacon, jabá, presunto de parma, pururuca de porco
20	Cervejas e vinhos	Cerveja, chopp, vinhos, espumante, saquê e drinks com essas bebidas
21	Outros	Conserva verdura/legume (chucrute, tomate seco, azeitonas, palmito, picles) e de cogumelos
		Frutas em conserva (goiabada, bananada, doce de abóbora, doces de frutas em calda, doces de frutas cristalizadas)
		Conserva leguminosa (milho em conserva, ervilha em conserva, pasta de amendoim)
		Molho de tomate
		Conserva peixe (sardinha em conserva, atum em conserva, salmão em conserva, bacalhau)
**Alimentos ultraprocessados**		
22	Bolacha salgada e salgadinho tipo chips	Biscoito salgado, rosquinha salgada, presuntinho, salgadinho de pacote (batata chips, salgadinho de bacon, amendoim apimentado, pipoca light)
23	Bolachas e panificados doces	Rosquinha, bolacha recheada, biscoito de polvilho doce, biscoito tipo *waffle* e outros
		Pães doces, rocamboles, sonho, panetone, muffin, croissant com recheio doce
24	Carnes embutidas	Hambúrguer de carne bovina ou peixe, *steak* de frango, salsicha, linguiça, mortadela, salame, presunto, peito de peru, patês com carne, calabresa
25	Guloseimas	Sorvetes, picolés, sorvete na casquinha, *milk shake*, geladinho, sorvete a base de iogurte
		Chocolate, bala e outras guloseimas (tablete de chocolate ao leite ou amargo ou branco, achocolatados em pó, goma de mascar, bala de caramelo, bala de coco, jujuba)
		Outros doces ultraprocessados (marshmallow, doce de leite, brigadeiro, queijadinha, geleia, barra de cereal, marrom glacê, doces light e diet, maria mole, quebra queixo, doce de amendoim)
26	Refrigerantes	Refrigerantes (incluindo light ou diet)
27	Pães, salgados e lanches tipo *fast food*	Lanches do tipo f*ast food* (Sanduíches variados com hambúrguer, cachorro-quente, wrap, x-tudo, x-*egg*, x- frango)
		Pão de hambúrguer, bisnaguinha, pão de milho, pão de centeio, pão de alho, pães light e diet, torradas
		Sanduíches com recheio de alimentos ultraprocessados (misto quente, pão com mortadela, sanduíche de salame)
		Salgados (pão de batata, coxinha, esfirra, croquete, pastel de forno, bolinha de queijo)
		Pizza salgada ou doce
28	Iogurtes e bebidas lácteas	Leite com sabor, fermentado ou achocolatado, bebida achocolatada, café tipo cappuccino, iogurte com sabor, concentrado alimentar shake)
29	Suco artificial e outras bebidas não alcóolicas	Sucos e refrescos artificiais e bebidas não alcoólicas (leite de soja, bebida isotônica, chás prontos, cerveja e vinho sem álcool, bebida energética)
30	Pratos prontos ou semiprontos para consumo	Refeições prontas massa (yakissoba, canelone recheado, rondele recheado, lasanha pronta) e macarrão instantâneo
31	Outros	Creme de queijo, requeijão, queijo cremoso, margarina
		Cereais Matinais (granola, flocos de milho com açúcar, farinha láctea)
		Bebidas alcoólicas destiladas (cachaça, rum, vodca, whisky, conhaque, licor e drinks com essas bebidas)
		Molhos industrializados (ketchup, shoyo, mostarda, molho tártaro, molho para salada, maionese light) e creme de leite
		Suplementos (suplemento proteico, vitaminas, minerais, complemento alimentar, cevada em pó)

^a^ Estas preparações podem eventualmente conter ingredientes ultraprocessados.

O grupo de alimentos *in natura* ou minimamente processados inclui os itens obtidos diretamente de plantas ou de animais e alimentos que tenham sido submetidos a algum processo de remoção de partes não desejadas, secagem, pasteurização, congelamento, refinamento, fermentação, dentre outros, que não incluem a adição de substâncias ao alimento original^20^. São exemplos de alimentos incluídos nesse grupo: arroz e outros cereais, feijões, carnes, frutas e sucos naturais, hortaliças, raízes e tubérculos, ovos, massas, chás e café e farinhas. Foram incluídas ainda as preparações culinárias baseadas em um ou mais alimentos *in natura* ou minimamente processados, como preparações mistas de arroz, carnes e vegetais e sobremesas caseiras.

Alimentos processados são produtos industrializados baseados em alimentos *in natura* ou minimamente processados aos quais tenha sido adicionado um ou mais ingredientes, como sal ou açúcar, óleo, vinagre ou outra substância de uso culinário, como no caso de carnes salgadas, pães feitos de farinha, sal e água e queijos feitos de leite e sal^20^. Preparações que combinam mais de um alimento processado também foram incluídas nesse grupo, como sanduíches feitos com pão francês.

O terceiro grupo, dos alimentos ultraprocessados, inclui formulações industriais tipicamente desenvolvidas a partir de partes de alimentos ou de substâncias sintetizadas em laboratório, feitas de inúmeros ingredientes, como açúcares e xaropes, amidos refinados, óleos e gorduras, isolados proteicos, além de restos de animais de criação intensiva^20^. Ingredientes *in natura* ou minimamente processados representam porções reduzidas ou nulas na lista de ingredientes dos alimentos ultraprocessados. Com o objetivo de serem atrativas, utiliza-se combinações de flavorizantes, corantes, emulsificantes, espessantes e outros aditivos que modificam as características sensoriais. Nesse grupo estão pães ultraprocessados, bolachas e salgadinhos, embutidos, guloseimas (sorvetes, chocolates, balas), refrigerantes, refeições prontas para o consumo ou congeladas, lanches do tipo *fast food*, bebidas lácteas, e sucos artificiais.

### Análise de Dados

O consumo alimentar individual foi ajustado para variabilidade intrapessoal, utilizando o método *Multiple Source Method* (MSM)^22,23^. A partir dessas estimativas de consumo habitual ajustado, foi calculado o percentual médio de calorias provenientes de cada um dos grupos de alimentos para o conjunto da população brasileira e de acordo com categorias de raça/cor da pele, que correspondem à autodeclaração da população entrevistada, a partir da escolha de uma das cinco opções: branca, preta, parda, indígena ou amarela. O percentual calórico dos grupos de alimentos por categoria de raça/cor da pele foi estimado através de modelos de regressão linear brutos e ajustados para sexo, faixa etária (adolescente, adulto e idoso), quintis de renda familiar mensal *per capita* em reais, quintis de escolaridade em anos completos ajustados para idade, macrorregião geográfica (Centro-Oeste, Nordeste, Norte, Sudeste e Sul) e área de residência (urbana ou rural).

Os cálculos levaram em conta o delineamento amostral complexo da pesquisa e seus fatores de expansão, que possibilitam a extrapolação dos resultados para toda a população brasileira. As estimativas e os respectivos intervalos de confiança (IC95%) são apresentados para as categorias de raça/cor da pele para todos os grupos e subgrupos de alimentos. As análises foram realizadas no programa *Stata*® versão 14 (College Station, TX: StataCorp LP).

## RESULTADOS

Dentre os indivíduos que responderam ao módulo de consumo alimentar da POF, 41 não informaram raça/cor da pele e, dos demais, 44,9% eram autodeclarados pardos, 43,1% brancos, 10,8% pretos, 0,7% amarelos e 0,4% indígenas (Tabela 1).

**Tabela 1:** Distribuição da população brasileira e média de consumo calórico diário médio, segundo raça/cor da pele. Brasil, 2017–2018.

Raça/cor da pele	Frequência na amostra		Consumo calórico médio	
	%	IC95%	kcal/dia	IC95%
Parda	44,9	44,0–45,9	1.721,1	1.690,2–1.751,9
Branca	43,1	42,1–44,2	1.761,5	1.745,6–1.777,4
Preta	10,8	10,2–11,5	1.723,9	1.634,3–1.813,5
Amarela	0,7	0,5–0,9	1.756,9	1.742,1–1.771,7
Indígena	0,4	0,3–0,6	1.713,9	1.618,1–1.809,6
**Total**	**100**	-	**1.754,6**	**1.743,5–1.765,8**

O consumo calórico médio da população brasileira foi de 1.754,6 kcal/dia, variando de 1.713,9 kcal/dia entre os indígenas até 1.761,5 kcal/dia entre a população de cor da pele branca (Tabela 1). Pardos, pretos e amarelos consumiram, em média, 1721,1 kcal, 1.723,9 kcal e 1.756,9 kcal/dia, respectivamente. A contribuição calórica de cada um dos três grandes grupos de alimentos e preparações culinárias para todo o Brasil e estratificada por raça/cor da pele é apresentada na Figura. Nacionalmente, esse consumo foi caracterizado pela participação majoritária de alimentos *in natura* e minimamente processados, que corresponderam a mais de 68% das calorias diárias. Na sequência, aparecem alimentos ultraprocessados (18,2%) e alimentos processados (13,2%).

**Figura:** Contribuição calórica média diária (% kcal/dia) dos três grupos de alimentos, segundo características do processamento industrial por raça/cor da pele de indivíduos com idade igual ou superior a 10 anos. Análises brutas e ajustadas para sexo, idade, renda, escolaridade, macrorregião e área de residência. Brasil 2017–2018.

De acordo com as análises brutas, o consumo de alimentos *in natura* e minimamente processados foi menor para amarelos [66,0% (IC95% 62,4–69,6)] e brancos [66,6% (IC95% 66,1–67,1)], enquanto contribuiu com aproximadamente 70% das calorias consumidas pela população de cor da pele preta [69,8% (IC95% 68,9–70,8)] e parda [70,2% (IC95% 69,7–70,7)]. A contribuição calórica relativa desse grupo na alimentação de indígenas foi de 68,6% (IC95% 64,6–72,6), não apresentando diferença estatística em relação às demais.

Em relação aos alimentos processados, não foram observadas diferenças estatísticas entre as categorias de cor da pele parda [13,1% (IC95% 12,8–13,5)], preta [13,5% (IC95% 12,9–14,2)], branca [13,2% (IC95% 12,9–13,5)] e indígena [13,6% (IC95% 11,1%16.2%)]. O menor consumo de alimentos desse grupo foi observado na população de pele amarela, cuja participação calórica foi de 9,2% (IC95% 7,2%–11,1%), significativamente diferente das demais categorias.

Já os alimentos ultraprocessados tiveram menor participação na alimentação de pretos [16,6% (IC95% 15,6–17,6)] e pardos [16,6% (IC95% 16,2–17,1)], ligeiramente inferior ao consumo dos indígenas, com 17,8% (IC95% 14,6–21,0). Por outro lado, as maiores contribuições foram para a população branca, com 20,1% (IC95% 19,6–20,6), e amarelos, com 24,5% (IC95% 20,0–29,1).

Após o ajuste para variáveis socioeconômicas e demográficas, houve diminuição das diferenças observadas entre as categorias de raça/cor da pele nos três grupos de alimentos e preparações culinárias, sendo mais expressiva no grupo de alimentos ultraprocessados.

Ainda assim, apesar da redução na magnitude das diferenças, as análises ajustadas apontam que brancos [67,9% (IC95% 67,4–68,3)] foram os que consumiram menos alimentos *in natura* e minimamente processados, seguidos por amarelos [68,0% (IC95% 65,0–71,1)] e indígenas [68,0% (IC95% 64,6–71,4)]. O maior consumo entre pretos [69,1% (IC95% 68,2–70,0)] e pardos [69,1% (IC95% 68,6–69,6)] permaneceu após ajuste. A diferença entre ambos, pretos e pardos, em relação aos brancos diminuiu de mais de três pontos percentuais (pp) para 1,2 pp.

A participação de alimentos processados continuou sendo aproximadamente 4 pp menor entre a população de cor da pele amarela [9,4% (IC95% 7,3–11,4)] em relação aos pardos [13,0% (IC95% 12,7–13,4)], pretos [13,2% (IC95% 12,5–13,8)] e brancos [13,5% (IC95% 13,1–13,8)] após o ajuste. Indígenas apresentaram a maior média de alimentos desse grupo, porém, com amplo intervalo de confiança [13,4% (IC95% 10,8–16,0)].

Após ajuste, o consumo de alimentos ultraprocessados diminuiu aproximadamente 2 pp para as duas categorias de raça/cor da pele que apresentaram as maiores médias: brancos [18,6% (IC95% 18,2–19,1)] e amarelos [22,4% (IC95% 18,6–26,2)]. Por outro lado, houve um aumento de aproximadamente 1 pp na média de consumo de pretos [17,7% (IC95% 16,8–18,6)] e pardos [17,8% (IC95% 17,3–18,3)]. O ajuste aumentou a contribuição calórica desse grupo de alimentos na dieta de indígenas [18,6% (IC95% 15,6–21,6)], o que fez com que as diferenças em relação aos demais grupos diminuíssem.

As estimativas brutas e ajustadas para cada um dos 31 subgrupos são apresentadas nas tabelas a seguir. Abaixo são descritos os principais resultados das análises, após ajuste. De maneira geral, para a maior parte dos itens de alimentos e preparações culinárias não houve diferença no consumo da população de cor da pele amarela e dos indígenas, em relação às demais categorias.

Conforme apresentado na Tabela 2, no grupo de alimentos *in natura* e minimamente processados, o arroz foi responsável pela maior contribuição energética para todas as categorias de raça/cor da pele, entretanto o consumo foi maior entre pretos [12,0% (IC95% 11,5–12,5)] e pardos [11,8% (IC95% 11,6–12,1)], quando comparados aos brancos [11,3% (IC95% 11,0–11,5)]. Para os demais grupos não houve diferença estatística, apesar de a categoria de cor da pele amarela [12,7% (IC95% 10,7–14,7)] apresentar a maior média para a participação desse item na dieta.

**Tabela 2:** Contribuição calórica média diária (% kcal/dia) de alimentos *in natura* e minimamente processados, de acordo com raça/cor da pele. Brasil, 2017–2018.

Raça/cor	Brutas	Ajustadas^a^	Brutas	Ajustadas^a^	Brutas	Ajustadas^a^	Brutas	Ajustadas^a^
	**Arroz**		**Carnes de boi e de porco**		**Preparações mistas^b^**		**Feijão**	
Parda	12,3 (12,1–12,6)	11,8 (11,6–12,1)	10,1 (9,9–10,4)	10,2 (10,0–10,4)	8,2 (7,9–8,5)	8,4 (8,1–8,7)	8,0 (7,8–8,2)	7,8 (7,6–8,0)
Branca	10,6 (10,4–10,9)	11,3 (11,0–11,5)	10,2 (10,0–10,5)	10,1 (9,9–10,4)	8,6 (8,3–8,9)	8,4 (8,1–8,8)	6,6 (6,4–6,8)	7,0 (6,8–7,2)
Preta	12,6 (12,1–13,1)	12,0 (11,5–12,5)	10,3 (9,8–10,8)	10,4 (9,9–10,9)	8,3 (7,6–9,0)	8,4 (7,8–9,0)	8,5 (8,1–8,9)	8,0 (7,6–8,4)
Amarela	11,6 (9,6–13,5)	12,7 (10,7–14,7)	8,8 (7,1–10,5)	9,1 (7,5–10,6)	10,5(7,9–13,0)	10,1 (7,6–12,6)	5,6 (4,6–6,6)	6,6 (5,6–7,5)
Indígena	11,4 (9,8–13,0)	11,3 (9,7–12,9)	10,4 (8,6–12,1)	10,6 (8,8–12,4)	7,6 (5,7–9,5)	7,5 (5,7–9,4)	6,7 (4,3–9,0)	6,6 (4,1–9,2)
	**Carnes de aves**		**Frutas e suco de frutas natural**		**Sobremesas caseiras^c^**		**Cafés e chás**	
Parda	6,8 (6,6–6,9)	6,5 (6,4–6,7)	4,4 (4,3–4,5)	4,7 (4,6–4,9)	3,0 (2,9–3,1)	3,1 (3,0–3,2)	2,3 (2,2–2,4)	2,4 (2,3–2,5)
Branca	5,9 (5,8–6,0)	6,2 (6,0–6,3)	5,2 (5,0–5,3)	4,8 (4,7–4,9)	3,4 (3,2–3,6)	3,2 (3,1–3,4)	2,6 (2,5–2,7)	2,5 (2,4–2,6)
Preta	6,4 (6,2–6,7)	6,3 (6,0–6,5)	4,6 (4,3–4,9)	4,8 (4,6–5,1)	2,9 (2,7–3,2)	3,1 (2,8–3,3)	2,4 (2,3–2,6)	2,4 (2,3–2,6)
Amarela	5,6 (4,7–6,6)	6,1 (5,2–7,0)	5,8 (4,5–7,2)	5,1 (3,8–6,4)	2,4 (1,6–3,2)	2,3 (1,4–3,1)	1,8 (1,3–2,3)	1,8 (1,2–2,3)
Indígena	6,6 (5,0–8,1)	6,4 (4,9–8,0)	4,2 (3,2–5,2)	4,3 (3,4–5,1)	3,4 (2,2–4,6)	3,5 (2,3–4,7)	2,3 (1,7–2,8)	2,2 (1,7–2,8)
	**Tubérculos e raízes**		**Macarrão**		**Verduras e legumes**		**Ovos**	
Parda	2,0 (1,9–2,1)	2,1 (2,0–2,2)	2,1 (2,0–2,2)	2,1 (2,0–2,1)	1,2 (1,2–1,3)	1,4 (1,3–1,4)	1,6 (1,5–1,7)	1,5 (1,5–1,6)
Branca	2,2 (2,1–2,3)	2,1 (2,0–2,2)	2,0 (1,9–2,2)	2,0 (1,9–2,2)	1,7 (1,6–1,7)	1,5 (1,4–1,5)	1,5 (1,4–1,6)	1,6 (1,5–1,7)
Preta	2,1 (1,9–2,3)	2,2 (2,0–2,4)	2,1 (1,9–2,3)	2,1 (1,9–2,3)	1,3 (1,2–1,4)	1,4 (1,3–1,5)	1,5 (1,3–1,6)	1,4 (1,3–1,5)
Amarela	1,7 (1,2–2,1)	1,6 (1,1–2,0)	1,8 (1,1–2,5)	2,0 (1,3–2,7)	2,0 (1,5–2,5)	1,7 (1,2–2,2)	1,7 (0,7–2,7)	1,8 (0,7–2,8)
Indígena	2,9 (0,9–5,0)	3,0 (0,9–5,1)	2,0 (1,4–2,6)	2,0 (1,4–2,6)	1,3 (0,9–1,7)	1,4 (1,0–1,8)	1,3 (0,9–1,7)	1,3 (0,9–1,7)
	**Peixes**		**Milho, aveia e trigo (excluindo farinhas)**		**Farinha de mandioca**		**Outros^d^**	
Parda	1,7 (1,6–1,8)	1,4 (1,3–1,5)	1,5 (1,4–1,5)	1,2 (1,1–1,3)	1,9 (1,8–2,0)	1,4 (1,3–1,5)	3,1 (2,9–3,3)	3,0 (2,8–3,1)
Branca	1,0 (0,9–1,1)	1,3 (1,2–1,4)	0,9 (0,8–1,0)	1,2 (1,1–1,3)	0,8 (0,8–0,9)	1,3 (1,2–1,4)	3,2 (3,0–3,4)	3,3 (3,1–3,5)
Preta	1,3 (1,1–1,5)	1,3 (1,1–1,4)	1,3 (1,1–1,5)	1,2 (1,0–1,3)	1,6 (1,4–1,8)	1,5 (1,3–1,7)	2,6 (2,4–2,9)	2,7 (2,5–3,0)
Amarela	1,3 (0,4–2,1)	1,4 (0,6–2,2)	0,6 (0,3–0,8)	1,1 (0,8–1,3)	1,4 (0,8–2,1)	1,7 (1,0–2,4)	3,5 (2,2–4,8)	3,2 (1,9–4,5)
Indígena	2,4 (1,2–3,5)	2,2 (1,3–3,2)	1,0 (0,5–1,4)	0,9 (0,5–1,3)	1,9 (0,9–2,8)	1,6 (1,0–2,1)	3,4 (1,8–4,9)	3,2 (1,9–4,5)

^a^ Ajuste para sexo, idade, renda, escolaridade, área de residência e região do país.

^b^ Preparações mistas que incluem arroz, carnes de qualquer origem e outros vegetais.

^c^ Inclui bolos, pães, tortas e outras sobremesas doces como bolos simples, ambrosia, cocada, curau, arroz doce, banana caramelizada, cajuzinho, quindim, pudim de leite, olho de sogra, delícia de abacaxi, bolo de cenoura com recheio de chocolate.

^d^ Inclui frutos do mar, outras carnes, iogurte natural, outras farinhas, leite, miúdos, pães e tortas salgadas, outras leguminosas, nozes, fungos, óleo e azeite, manteiga, açúcar, banha, leite de coco, nata e outros ingredientes culinários.

Um padrão semelhante foi observado para o consumo de feijão, cuja contribuição energética foi maior entre pretos [8,0% (IC95% 7,6–8,4)] e pardos [7,8% (IC95% 7,6–8,0)], em comparação à população branca [7,0% (IC95% 6,8–7,2)]. Indígenas [6,6% (IC95% 4,1–9,2)] e amarelos [6,6% (IC95% 5,6–7,5)] apresentaram consumo semelhante e menor que os demais grupos.

Em relação às carnes de aves, indivíduos pardos [6,5% (IC95% 6,4–6,7)] tiveram maior consumo que brancos [6,2% (IC95% 6,0–6,3)] e as demais categorias não apresentaram diferença significativa.

Os demais itens do grupo não apresentaram diferenças estatísticas entre as categorias de cor da pele, porém observou-se que preparações mistas que combinam arroz, carnes e vegetais aparecem com mais frequência para indivíduos de pele amarela (10,1%) em relação aos demais, sendo que os indígenas foram aqueles com menor consumo desses alimentos (7,5%). Por outro lado, os indígenas foram aqueles com maior consumo absoluto de peixes (2,2%) e raízes e tubérculos (3,0%). Amarelos (1,7%) e brancos (1,5%) possuem consumo ligeiramente maior de legumes e verduras em relação aos demais grupos.

Em relação aos subgrupos de alimentos processados, as preparações com maior contribuição energética para todas as categorias de raça/cor da pele foram pão francês e sanduíches (também feitos com pão francês), como mostrado na Tabela 3. O consumo desse grupo de alimentos foi caracterizado pela menor contribuição calórica para a população de pele amarela [7,5%, (IC95% 6,3–8,7)], com diferença estatística em relação às demais, e maior consumo absoluto pela população indígena [11,2% (IC95% 8,8–13,7)]. Não foram encontradas diferenças no consumo de indivíduos brancos, pardos e pretos e as médias ficaram entre 10,4% e 10,6%. A população branca apresentou consumo de queijos processados [0,9% (IC95% 0,8–1,0)], com diferenças significativas em relação aos pretos [0,6% (IC95% 0,5–0,7)] e pardos [0,7% (IC95% 0,6–0,7)].

**Tabela 3:** Contribuição calórica média diária (% kcal/dia) de alimentos processados, de acordo com raça/cor da pele. Brasil, 2017–2018.

Raça/cor	Brutas	Ajustadas^a^	Brutas	Ajustadas^a^	Brutas	Ajustadas^a^	Brutas	Ajustadas^a^
	**Pão francês e sanduíches^b^**		**Cervejas e vinhos**		**Queijos**		**Carnes salgadas/secas/ defumadas/curadas**	
Parda	10,7 (10,4–10,9)	10,4 (10,1–10,7)	1,0 (0,9–1,2)	1,2 (1,0–1,4)	0,5 (0,5–0,6)	0,7 (0,6–0,7)	0,5 (0,5–0,6)	0,4 (0,3–0,5)
Branca	10,2 (9,9–10,5)	10,6 (10,3–10,9)	1,3 (1,2–1,4)	1,1 (1,0–1,2)	1,1 (1,0–1,2)	0,9 (0,8–1,0)	0,3 (0,2–0,3)	0,4 (0,4–0,5)
Preta	11,1 (10,5–11,8)	10,6 (10,0–11,2)	1,0 (0,9–1,2)	1,2 (1,0–1,3)	0,5 (0,4–0,6)	0,6 (0,5–0,7)	0,6 (0,4–0,8)	0,5 (0,4–0,7)
Amarela	6,9 (5,6–8,2)	7,5 (6,3–8,7)	0,6 (0,2–1,1)	0,3 (0,0–0,8)	1,3 (0,0–2,7)	1,0 (0,0–2,3)	0,3 (0,0–0,5)	0,4 (0,2–0,6)
Indígena	11,5 (8,8–14,3)	11,2 (8,8–13,7)	0,8 (0,2–1,4)	0,9 (0,3–1,5)	0,9 (0,2–1,5)	0,9 (0,3–1,5)	0,3 (0,0–0,5)	0,2 (0,0–0,4)
	**Outros^c^**							
Parda	0,4 (0,3–0,4)	0,4 (0,3–0,4)						
Branca	0,4 (0,4–0,5)	0,4 (0,3–0,4)						
Preta	0,3 (0,2–0,3)	0,3 (0,2–0,4)						
Amarela	0,2 (0,0–0,3)	0,1 (0,0–0,3)						
Indígena	0,2 (0,0–0,4)	0,2 (0,0–0,4)						

^a^ Ajuste para sexo, idade, renda, escolaridade, macrorregião e área de residência.

^b^ Inclui somente sanduíches de pão de francês.

^c^ Conserva de verdura/legume, doce de fruta, nozes processadas, conserva leguminosa, molho de tomate, conserva de peixe.

O consumo de cervejas e vinhos foi mais baixo para as populações de pele amarela [0,3% (IC95% 0,0–0,8)] – significativamente menor do que para as demais – e indígena [0,9% (IC95% 0,3–1,5)]. Para os demais itens que compõem o grupo de alimentos processados, não foram observadas diferenças significativas entre as categorias de raça/cor da pele.

No grupo de alimentos ultraprocessados, apesar de não haver diferenças significativas entre as categorias de raça/cor da pele para nenhum dos itens após o ajuste das análises, observou-se que os alimentos com maior contribuição calórica foram os lanches prontos para consumo, que inclui sanduíches do tipo *fast food*, salgados fritos e assados etc., conforme apresentado na Tabela 4. Apesar da ausência de significância estatística em relação às demais categorias, indivíduos de cor da pele amarela foram aqueles com a maior média de consumo desse grupo de alimentos, que contribuiu com 6,8% (IC95% 4,9–8,6) das calorias, enquanto indígenas apresentaram o menor consumo, com 3,7% (IC95% 2,3–5,1). As demais categorias são intermediárias no consumo e a população branca [5,8% (IC95% 5,5–6,1)] consumiu ligeiramente mais alimentos ultraprocessados do que pretos [5,3% (IC95% 4,5–6,0)] e pardos [5,4% (IC95% 4,9–5,8)].

**Tabela 4:** Contribuição calórica média diária (% kcal/dia) de alimentos ultraprocessados, de acordo com raça/cor da pele. Brasil, 2017–2018.

Raça/cor	Brutas	Ajustadas^a^	Brutas	Ajustadas^a^	Brutas	Ajustadas^a^	Brutas	Ajustadas^a^
	**Pães, salgados e lanches do tipo fast food^b^**		**Bolacha salgada e salgadinhos tipo chips**		**Bolachas e panificados doces**		**Refrigerantes**	
Parda	4,6 (4,1–5,0)	5,4 (4,9–5,8)	2,8 (2,7–2,9)	2,7 (2,5–2,8)	2,2 (2,1–2,3)	2,2 (2,1–2,3)	1,4 (1,4–1,5)	1,5 (1,4–1,6)
Branca	6,8 (6,4–7,1)	5,8 (5,5–6,1)	2,6 (2,4–2,7)	2,7 (2,6–2,8)	2,2 (2,1–2,4)	2,2 (2,1–2,3)	1,6 (1,5–1,6)	1,4 (1,4–1,5)
Preta	4,7 (3,9–5,4)	5,3 (4,5–6,0)	2,8 (2,5–3,0)	2,8 (2,5–3,0)	2,0 (1,8–2,2)	2,1 (1,8–2,3)	1,4 (1,2–1,5)	1,5 (1,3–1,6)
Amarela	8,3 (6,4–10,2)	6,8 (4,9–8,6)	2,6 (1,7–3,6)	2,7 (1,8–3,6)	2,0 (1,2–2,8)	2,2 (1,5–2,9)	1,9 (1,3–2,6)	1,9 (1,3–2,6)
Indígena	3,2 (1,4–3,4)	3,7 (2,3–5,1)	3,0 (1,9–4,0)	2,9 (1,9–3,9)	2,9 (1,5–4,3)	3,0 (1,5–4,4)	2,1 (1,1–3,1)	2,2 (1,3–3,0)
	**Guloseimas^c^**		**Carnes embutidas**		**Iogurtes e bebidas lácteas**		**Pratos prontos ou semiprontos^d^**	
Parda	0,9 (0,9–1,0)	1,1 (1,0–1,2)	1,3 (1,2–1,4)	1,3 (1,2–1,4)	0,9 (0,8–1,0)	1,0 (0,9–1,1)	0,9 (0,8–1,0)	0,9 (0,8–1,1)
Branca	1,4 (1,3–1,5)	1,2 (1,1–1,4)	1,3 (1,2–1,4)	1,3 (1,2–1,4)	1,3 (1,2–1,4)	1,1 (1,0–1,2)	1,2 (1,0–1,3)	1,1 (1,0–1,2)
Preta	1,1 (0,9–1,3)	1,2 (1,1–1,4)	1,3 (1,2–1,4)	1,3 (1,1–1,4)	0,8 (0,7–1,0)	0,9 (0,8–1,1)	0,9 (0,6–1,1)	0,9 (0,7–1,2)
Amarela	1,9 (0,6–3,2)	1,6 (0,3–2,8)	1,7 (0,9–2,5)	1,8 (1,0–2,5)	1,0 (0,5–1,5)	0,8 (0,2–1,3)	0,6 (0,0–1,2)	0,5 (0,0–1,1)
Indígena	0,9 (0,3–1,5)	1,0 (0,3–1,6)	1,4 (0,7–2,2)	1,4 (0,8–2,1)	1,8 (0,1–3,5)	1,9 (0,3–3,4)	0,8 (0,2–1,3)	0,8 (0,2–1,4)
	**Suco artificial e outras bebidas não alcoólicas**		**Outros^e^**					
Parda	1,2 (1,1–1,2)	1,2 (1,1–1,3)	0,5 (0,4–0,5)	0,5 (0,5–0,6)				
Branca	1,2 (1,1–1,3)	1,2 (1,1–1,3)	0,7 (0,6–0,8)	0,6 (0,5–0,7)				
Preta	1,3 (1,2–1,5)	1,4 (1,2–1,5)	0,4 (0,3–0,5)	0,5 (0,4–0,6)				
Amarela	3,3 (0,0–6,7)	3,2 (0,0–6,5)	1,4 (0,4–2,4)	1,3 (0,4–2,3)				
Indígena	1,0 (0,3–1,8)	1,0 (0,3–1,8)	0,7 (0,0–1,4)	0,7 (0,0–1,5)				

^a^ Ajuste para sexo, idade, renda, escolaridade, macrorregião e área de residência.

^b^ Inclui hambúrgueres, cachorro-quente, pizzas, salgados fritos e assados, e semelhantes.

^c^ Inclui balas, confeitos, chocolates, sorvete e semelhantes.

^d^ Pratos de massa ou de carne congelados, macarrão instantâneo e sopas em pó,

^e^ Inclui creme de leite, requeijão, queijo cremoso, margarina, cereais matinais, bebidas alcoólicas destiladas e drinks com essas bebidas), molhos industrializados e suplementos.

Para a população de cor da pele amarela, destacam-se as maiores estimativas pontuais de consumo de lanches e pães ultraprocessados (6,8%), suco artificial e outras bebidas não alcóolicas (3,2%), embutidos (1,8%) e guloseimas (1,6%), enquanto indígenas consomem menos calorias provenientes de pães ultraprocessados (3,7%), mas apresentam um percentual ligeiramente maior de consumo de refrigerantes (1,9%) e bebidas lácteas (1,9%) em relação às demais categorias.

## DISCUSSÃO

Nossos resultados revelam que pretos e pardos corresponderam à categoria com maior consumo de alimentos *in natura* e minimamente processados, enquanto a população de pele branca e amarela consumiu mais alimentos ultraprocessados, quando comparada às demais. Amarelos também apresentaram o menor consumo de alimentos processados.

Recomendações alimentares atuais, incluindo o Guia Alimentar para a População Brasileira, indicam que uma alimentação saudável deve ser baseada em alimentos *in natura* e minimamente processados, com quantidade limitada de alimentos processados e que se evite o consumo de ultraprocessados^14,24^. Apesar de as categorias de raça/cor da pele parda, preta e indígena apresentarem um consumo convergente ao recomendado, as mudanças observadas nas análises com e sem ajuste para variáveis de confusão indicam que essas diferenças podem estar associadas a condições socioeconômicas e demográficas. O consumo alimentar é resultado da interação de distintos fatores, sendo influenciado por características culturais, disponibilidade e acesso a alimentos. Nas nossas análises, parte das variações entre as categorias de raça/cor da pele foi explicada por características socioeconômicas e demográficas, demonstrado por meio da redução na magnitude das diferenças nos modelos ajustados.

Em relação aos alimentos *in natura* e minimamente processados, nas análises brutas, brancos e amarelos apresentaram maiores médias de consumo de frutas, verduras e legumes, diferença que não permaneceu após ajuste. Por outro lado, as diferenças mais expressivas que se mantiveram após ajuste foram observadas nas médias de consumo de arroz e de feijão, itens para os quais as categorias de cor da pele parda e preta apresentaram os maiores valores com diferença estatística em relação às demais. Apesar de os nossos resultados indicarem que pretos e pardos apresentaram maior adesão às recomendações do Guia Alimentar para a População Brasileira quanto à maior participação relativa de preparações à base de arroz e feijão, isso não representa maior qualidade global da alimentação, já que outros alimentos como frutas, legumes e verduras não apresentaram a mesma associação. Outros estudos que incluíram consumo alimentar nas análises também encontraram que pretos e pardos apresentam menor consumo recomendado ou regular de frutas e hortaliças e maior consumo de feijão^7,10,11,13^. A perda de significância estatística foi particularmente importante para alimentos ultraprocessados, grupo em que nenhuma das diferenças observadas nas análises brutas permaneceu após ajuste.

Há na literatura uma consistência em estudos concluindo que a renda é um importante fator associado ao consumo alimentar, o que por sua vez está relacionado ao acesso influenciado pelo preço dos alimentos^25^. Desde os anos 1990 é observada uma tendência na redução dos preços de alimentos ultraprocessados no Brasil, mesmo que ainda sejam mais caros do que os alimentos *in natura*, minimamente processados e processados. O baixo custo de modo geral de grãos como arroz e feijão pode contribuir para que a alimentação tradicional dos brasileiros ainda seja mais acessível^28^.

Diante dessas evidências, a interpretação dos resultados sobre o consumo alimentar precisa ser realizada à luz dos processos sociais ocorridos no Brasil, um país com histórico escravagista, cujas consequências são marcadas por desigualdades diretamente atreladas a questões étnico-raciais^29^. De acordo com dados oficiais do IBGE, pretos e pardos apresentam maiores proporções de indivíduos analfabetos e trabalhando na informalidade e menor rendimento médio, em comparação com a população branca^30^. De maneira similar, as populações indígenas sofrem com a marginalização, a pobreza e a discriminação^31,32^. Além disso, dados da POF 2017–2018 também revelaram que entre os grupos de pele preta e parda se observa maior frequência de insegurança alimentar^33^, resultado que indica que, mesmo apresentando maior consumo relativo de arroz e feijão em comparação a grupos mais privilegiados socioeconomicamente, as condições de alimentação da população preta e parda refletem a condição de vulnerabilidade social a que está sujeita. Desse modo, as desigualdades observadas no consumo podem ser atribuídas não apenas a características dos grupos raciais, mas às suas condições sociais, econômicas e demográficas.

Devido à complexidade das relações raciais no Brasil, optou-se por utilizar neste estudo as cinco categorias de raça ou cor definidas pelo IBGE para coleta de dados da população brasileira, visto que a cor da pele no país é um importante marcador social das desigualdades^34^. Além disso, a utilização das cinco categorias visibiliza possíveis iniquidades a grupos raciais mais vulneráveis.

Apesar de ser um inquérito de representatividade nacional, a POF não é delineada para ser representativa dos grupos definidos por raça ou cor da pele e, assim, o tamanho amostral para algumas categorias – especificamente amarelos e indígenas – não contempla de forma equânime os grupos populacionais e não apresenta poder estatístico suficiente para que possíveis diferenças possam ser identificadas, o que se configura como uma limitação constante para avaliação da população brasileira.

Além dessa característica limitante própria dos inquéritos por amostragem, muitos estudos optam por não incluir os grupos de menor tamanho nas análises^8,10,13^.Alguns autores utilizam a categoria *negros*, criada a partir da combinação dos grupos de cor da pele preta e parda, como um único estrato de análise, e outros utilizam uma abordagem binária, comparando dois grandes grupos definidos como *brancos* e *não-brancos,* tratando branco como o normativo e invisibilizando as diferenças entre as demais categorias de raça/cor da pele^9,12^.

Ainda que por um lado tenham aparecido consistentes semelhanças no consumo alimentar de pretos e pardos e, por outro, diferenças estatísticas não tenham sido observadas para alguns itens, devido aos amplos intervalos de confiança para indígenas e amarelos, foi possível descrever algumas variações importantes. O maior consumo de arroz e preparações mistas de arroz, carnes e leguminosas para a população amarela e de peixes, raízes e tubérculos para indígenas são exemplos que ilustram marcadores culturais da alimentação.

Nossos resultados evidenciam a marcante presença do binômio arroz com feijão na alimentação brasileira. Presente no país todo, não se sabe ao certo desde quando essa combinação está na mesa dos brasileiros, mas a partir do século XX sua presença é vista no cotidiano e em pratos típicos das diferentes regiões. Além disso, o feijão, também conhecido como “carne de pobre”, com arroz tem importância relevante principalmente nos lares de menor poder aquisitivo do país, considerado saudável pela sua combinação nutricional^35^.

É importante ressaltar ainda que as diferenças observadas podem variar em magnitude quando considerada a distribuição geográfica dos grupos pelo país e, desse modo, recomenda-se que análises futuras explorem também as desigualdades subnacionais.

Inquéritos populacionais e sistemas de informação em saúde têm incluído o campo raça/cor da pele em suas coletas, porém, além de muitas vezes apresentarem baixa frequência de preenchimento^3,36^, ainda é necessária a incorporação dos dados coletados em análises, como parte fundamental do processo de compreensão da situação epidemiológica da população brasileira. Estudos sobre desigualdades em comportamentos e prevalência de fatores de risco por raça/cor da população são escassos e, muitas vezes, essa informação aparece diluída em análises que incluem outras dimensões.

Algumas limitações metodológicas são comuns aos inquéritos que coletam dados de consumo alimentar, incluindo possível viés de aferição da dieta habitual, falha de memória do entrevistado e falta de precisão nas medidas de consumo. Para reduzir seus impactos nos resultados, foram utilizados questionários validados e a coleta contou com controle de qualidade, dentre outros procedimentos padrão de manipulação de dados^17,37^.

## CONCLUSÕES

Este estudo é um passo importante para o preenchimento de uma lacuna na literatura científica sobre a variação do consumo alimentar entre os grupos etnorraciais no Brasil. Encontrou-se diferenças na participação de alimentos *in natura* e minimamente processados e alimentos ultraprocessados na dieta da população brasileira, que aparecem associadas à posição socioeconômica dos indivíduos na sociedade que, de maneira geral, são desfavoráveis para pretos, pardos e indígenas. Portanto, iniciativas e políticas públicas para redução das desigualdades que afetam desproporcionalmente os grupos racializados precisam ocorrer concomitantemente àquelas que visam o incentivo do consumo de alimentos *in natura* e minimamente processados e à redução do consumo de alimentos ultraprocessados, cuja participação tem crescido na alimentação da população brasileira, com efeitos prejudiciais para a saúde de todos. Diante dessas evidências, esforços continuados devem ser feitos para que mais estudos incluam a descrição epidemiológica da população por raça/cor da pele, contribuindo para a saúde pública e para a compreensão e combate às iniquidades.
